# Analysis of COF-300 synthesis: probing degradation processes and 3D electron diffraction structure

**DOI:** 10.1107/S2052252524003713

**Published:** 2024-05-10

**Authors:** Laurens Bourda, Subhrajyoti Bhandary, Sho Ito, Christian R. Göb, Pascal Van Der Voort, Kristof Van Hecke

**Affiliations:** a XStruct, Department of Chemistry, Ghent University, Krijgslaan 281–S3, 9000Ghent, Belgium; b COMOC – Center for Ordered Materials, Organometallics and Catalysis – Department of Chemistry Ghent University Krijgslaan 281–S3 9000Ghent Belgium; c Rigaku Corporation, Haijima, Tokyo, Japan; d Rigaku Europe SE, Neu-Isenburg, Germany; Ben-Gurion University of the Negev, Israel

**Keywords:** 3D electron diffraction, 3DED, microcrystal electron diffraction, microED, covalent organic frameworks, Cambridge Structural Database, porous organic solids, crystallization and crystal growth

## Abstract

Although COF-300 is often used as an example to study the synthesis and structure of (3D) covalent organic frameworks (COFs), knowledge of the underlying synthetic processes is still fragmented. Here, an optimized synthetic procedure based on a combination of linker protection and modulation was applied. Using this approach, the influence of time and temperature on the synthesis of COF-300 was studied. Synthesis times that were too short produced materials with limited crystallinity and porosity, lacking the typical pore flexibility associated with COF-300. On the other hand, synthesis times that were too long could be characterized by loss of crystallinity and pore order by degradation of the tetrakis(4-aminophenyl)methane (TAM) linker used. The presence of the degradation product was confirmed by visual inspection, Raman spectroscopy and X-ray photoelectron spectroscopy (XPS). As TAM is by far the most popular linker for the synthesis of 3D COFs, this degradation process might be one of the reasons why the development of 3D COFs is still lagging compared with 2D COFs. However, COF crystals obtained via an optimized procedure could be structurally probed using 3D electron diffraction (3DED). The 3DED analysis resulted in a full structure determination of COF-300 at atomic resolution with satisfying data parameters. Comparison of our 3DED-derived structural model with previously reported single-crystal X-ray diffraction data for this material, as well as parameters derived from the Cambridge Structural Database, demonstrates the high accuracy of the 3DED method for structure determination. This validation might accelerate the exploitation of 3DED as a structure determination technique for COFs and other porous materials.

## Abstract 2

Detailed analysis of the influence of time and temperature on the synthesis of COF-300 showed partial linker degradation but still allowed 3D electron diffraction structure solution using optimized conditions.

## 1. Introduction

Covalent organic frameworks (COFs), pioneered by Yaghi and coworkers (Cote *et al.*, 2005 ▸), combine multiple highly desirable properties, making them an intriguing class of metal-free functional materials. Highly stable materials can be obtained, especially using Schiff base chemistry (Segura *et al.*, 2016 ▸). Furthermore, it is possible to tune and predict the 3D structures of these materials using the principles of reticular chemistry (Yaghi *et al.*, 2019 ▸). This allows the synthesis of highly porous and functionalized materials with known chemical compositions. Naturally, this has led to the usage of COFs in a whole range of applications, including gas storage, catalysis, sensor materials and energy storage (Thomas, 2010 ▸; Das *et al.*, 2017 ▸; Wang *et al.*, 2021 ▸; Shukla *et al.*, 2022 ▸; Xue *et al.*, 2023 ▸).

As the first imine COF to be reported (Uribe-Romo *et al.*, 2009 ▸) and one of the most researched COFs to date, COF-300 is often used as a benchmark material for improvement of the synthetic methodology and structural understanding (Wang *et al.*, 2023 ▸; Sun *et al.*, 2023 ▸). For example, the first report of single-crystal COFs used COF-300 as an example (Ma *et al.*, 2018*b* ▸). For this, large amounts of aniline were added as a modulator to grow single crystals of sufficient size for analysis by single-crystal X-ray diffraction (SCXRD). However, until now, only fragmented observations of the underlying synthetic processes and possible issues with COF-300 synthesis have been reported (Fischbach *et al.*, 2019 ▸; Wang *et al.*, 2023 ▸). Therefore, to truly establish COF-300 as a benchmark material for the study and improvement of COF synthesis, more extensive knowledge about the synthetic process is necessary.

Furthermore, it has proven difficult to extend the application of the aforementioned synthesis of single-crystal COFs to more challenging materials, with only a limited number of reports of novel 3D COFs since their inception in 2018 (Liang *et al.*, 2020 ▸; Gropp *et al.*, 2020 ▸; Zhou *et al.*, 2023 ▸; Yu *et al.*, 2023*b* ▸,*a* ▸) and a complete absence of 2D COFs. This observation can be explained by what is commonly referred to as ‘the crystallization problem’ (Haase & Lotsch, 2020 ▸; Bourda *et al.*, 2021 ▸). Essentially, this boils down to the high stability of COFs hampering their crystallinity. Therefore, the characterization of COFs via powder X-ray diffraction (PXRD) is more common by comparison with simulated model structures. However, due to the often weak, broad, overlapping and limited number of reflections, ambiguity in structure solution might persist (Bourda *et al.*, 2021 ▸; Huang *et al.*, 2021*c* ▸). Recently, 3D electron diffraction (3DED) has emerged as a promising technique to determine the atomic resolution structure of materials with very small crystal-sizes/crystallites (≤50 nm) that are not suitable for routine SCXRD analysis, where a minimum crystal size of around 50 µm is required (Gruene & Mugnaioli, 2021 ▸). Though already a well established technique for metal–organic frameworks (MOFs) (Huang *et al.*, 2021*a* ▸*c* ▸), only few COF structures have been studied using only 3DED until now. Moreover, 3DED data were mainly used as a starting point to build a structure model for comparison with PXRD data (Ding *et al.*, 2018 ▸; Ma *et al.*, 2018*a* ▸; Natraj *et al.*, 2022 ▸). In selected cases, data quality was sufficient to allow *ab initio* structure determination, albeit often with limited resolution (∼1.5 Å) (Zhang *et al.*, 2013 ▸), high *R* values (≥0.3) (Gao *et al.*, 2019 ▸; Zhou *et al.*, 2023 ▸) or low completeness (≤70%) (Sun *et al.*, 2019 ▸; Kang *et al.*, 2022 ▸). This can be partly explained by the still insufficient quality of most COFs, with very limited crystallinity, but also the strong interactions of electrons causing dynamical diffraction effects (Huang *et al.*, 2021*c* ▸; Gruene & Mugnaioli, 2021 ▸). Therefore, it is of paramount importance to confirm the accuracy of the technique, for example by comparing 3DED structures with SCXRD data, something which has been done for MOFs, zeolites and metal oxides, but is still lacking for COFs (Yun *et al.*, 2014 ▸; Wang *et al.*, 2018 ▸; Huang *et al.*, 2021*b* ▸). As a benchmark material which has already been studied with both SCXRD and 3DED, COF-300 would be an excellent candidate for such a study.

To enable the collection of high-quality 3DED data, the crystallinity of the COFs needs to be maximized. This can be reached by careful study of the COF formation kinetics and tweaking the conditions based on this knowledge, which is already quite developed for 2D COFs, as previously reviewed by us (Bourda *et al.*, 2021 ▸). For example, the usage of protected monomers has been shown to enhance COF quality, as the number of moieties available to react is limited by the slow deprotection of the monomer (Vitaku & Dichtel, 2017 ▸). Alternatively, the addition of monodentate aromatic amines like aniline has been shown to increase the crystallinity of 2D imine COFs (Yaghi *et al.*, 2019 ▸). However, even though single-crystal 3D imine COFs were obtained via a modulation process (Ma *et al.*, 2018*b* ▸), not much is known about the kinetics associated with 3D COF formation (Bourda *et al.*, 2021 ▸). Such a study would not only increase our knowledge on 3D COFs, but also open up a toolbox for tackling the even more pronounced crystallization problems for these 3D materials (Guan *et al.*, 2020 ▸).

**Figure 1:**
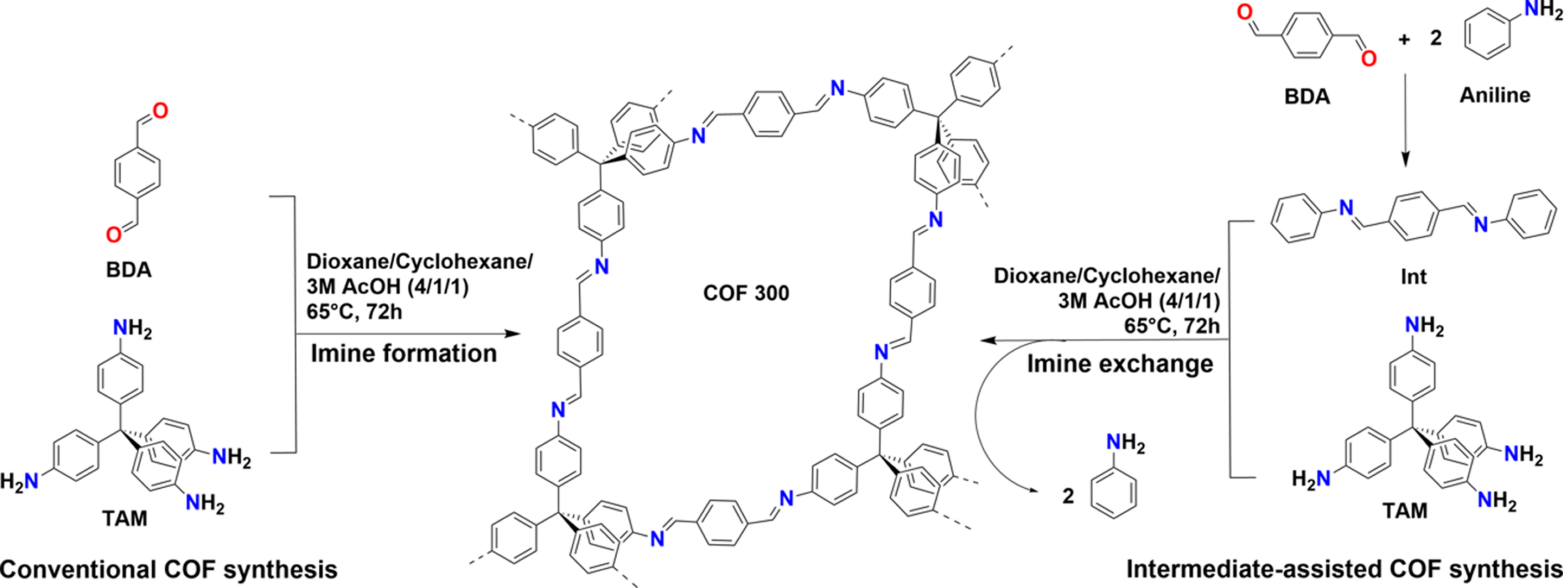
Scheme comparing the synthesis of COF-300 via the conventional route and the intermediate-assisted route.

Therefore, in this work we sought to increase our understanding of the COF-300 synthesis. The mild conditions described in the ‘ventilation vial’ procedure [65°C, no degassing (Chen *et al.*, 2019 ▸)] were taken as a starting point from which the synthesis of COF-300 was further optimized. Two of the previously described techniques, monomer protection and modulation, were combined in an ‘intermediate-assisted COF synthesis’ protocol (as presented alongside a conventional pathway in Fig. 1 ▸). By reacting the desired aldehyde monomer with equimolar amounts of aniline to the aldehyde functionalities, imine monomers [(1*E*,1′*E*)-1,1′-(1,4-phenylene)bis(*N*-phenylmethanimine), intermediate, Int] are obtained, which are then used for COF synthesis instead of the corresponding aldehydes. As a consequence, the COF synthesis is immediately determined by a combination of imine condensation, exchange and metathesis reactions, increasing the rate of error correction (Rowan *et al.*, 2002 ▸; Belowich & Stoddart, 2012 ▸; Ciaccia & Di Stefano, 2015 ▸). The protocol described has already been shown to work successfully for 2D COFs (Maia *et al.*, 2018 ▸; Zhao *et al.*, 2018 ▸; Kang *et al.*, 2022 ▸), was already employed by us for the synthesis of lanthanide grafted COF-300 (Bourda *et al.*, 2023 ▸), and was very recently even reported as a tool for the ultra-fast preparation of COF-300 (Wang *et al.*, 2023 ▸).

Based on the method developed, we studied the evolution of COF crystallinity and porosity over time, as well as the influence of temperature (65°C versus room temperature). Careful evaluation of these properties over time led to the first observation of linker degradation during the synthetic process, which can be detrimental for all COFs based on the tetrakis(4-aminophenyl)methane (TAM) linker if not properly accounted for. As the majority of 3D COFs reported to date use TAM (or a close derivate) as a linker, this might partially explain the increased difficulty observed in the synthesis of 3D COFs compared with 2D COFs (Guan *et al.*, 2020 ▸; Bourda *et al.*, 2021 ▸). Therefore, knowledge of this degradation process might help to close the gap in synthetic toolboxes between 3D and 2D COFs. Still, using the optimized conditions, full structure determination via state-of-the-art 3DED with satisfying resolution, completeness and *R* values was made possible. This was used for an in-depth analysis of the accuracy of structure determination with 3DED for COFs by comparison with the reported structure of COF-300 obtained via SCXRD (Ma *et al.*, 2018*b* ▸), as well as parameters derived from similar chemical functionalities found in the Cambridge Structural Database (CSD) (Groom *et al.*, 2016 ▸).

## 2. Results and discussion

**Figure 2:**
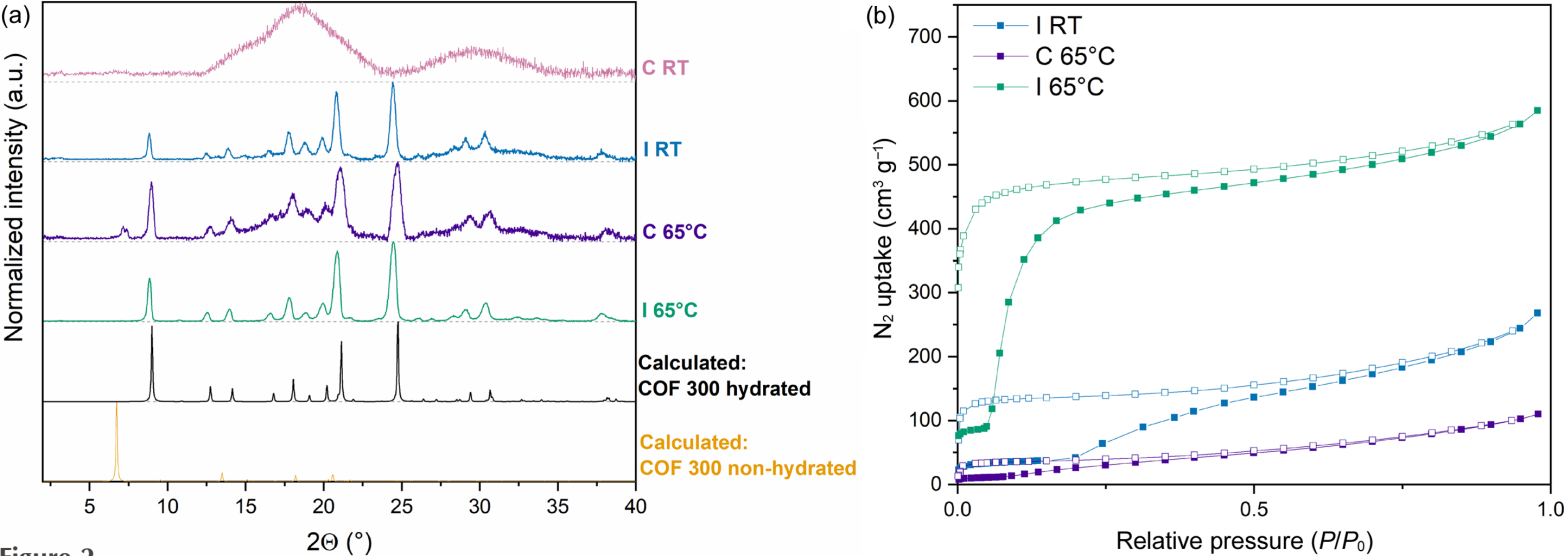
Normalized background-corrected (*a*) PXRD and (*b*) N_2_-sorption analysis of COF-300 synthesized via different approaches: intermediate-assisted synthesis at 65°C (green) and room temperature (blue), as well as the conventional synthesis at 65°C (purple) and room temperature (pink). No N_2_-sorption isotherm was measured for the conventional method at room temperature as the resulting material was completely amorphous. Calculated PXRD patterns based on the SCXRD structure of COF-300 (Ma *et al.*, 2018*b* ▸) (orange) and COF-300 hydrated (black) (Ma *et al.*, 2018*b* ▸) are included for comparison.

Firstly, Int was prepared by simply combining equimolar quantities of terephthalaldehyde (TA) and aniline and refluxing in methanol for 4 h before filtering (Scheme S1). Subsequently, to test the potential of the method, synthesis of COF-300 via the conventional method and the intermediate-assisted synthesis were compared. Therefore, four samples were prepared using identical conditions [1,4-dioxane/cyclohexane 4/1 *v*/*v* as the solvent, 0.4 ml acetic acid (3 mol l^−1^) as the catalyst, treated for 3 d in a 4 ml vial]. The only altered factor was the reaction temperature (65°C or room temperature) and the starting materials used: TA and TAM for the conventional process, TAM and Int for the intermediate-assisted synthesis. The resulting background-corrected PXRD patterns are presented in Fig. 2 ▸(*a*). Note from the good match between the calculated PXRD pattern for COF-300 hydrated and the experimental patterns, all samples formed the hydrated structure of COF-300. However, the conventional synthesis at room temperature (C RT) did not succeed in forming crystalline COF-300 with only amorphous material obtained. Additionally, it can be concluded that the crystallinity obtained for the 65°C synthesis is better for the intermediate-assisted synthesis (I 65°C) compared with the conventional sample (C 65°C) as shown by the lower peak intensity (visible by the low signal-to-noise ratio) and underlying amorphous band observed for the latter. Finally, though less pronounced than for C 65°C, the intermediate-assisted sample at room temperature (I RT) still shows some noise, indicating that the best crystallinity was obtained for sample I 65°C. Similar trends can be observed for the porosity of the studied materials. High porosity was observed for sample I 65°C with a Brunauer–Emmett–Teller (BET) surface area of around 1550 m^3^ g^−1^ and pore volume (*V*_p_) of 0.90 cm^3^ g^−1^ [Fig. 2 ▸(*b*)]. In contrast, the observed porosity for I RT and C 65°C are far lower with obtained BET surface areas of 150 and 50 m^2^ g^−1^, respectively, while corresponding *V*_p_ values were 0.21 and 0.80 cm^3^ g^−1^, respectively. The limited porosities of I RT and C 65°C illustrate that a small decrease in crystallinity can have a far more pronounced effect on the porosity of the material. Note that while considerably lower than I 65°C, the observed porosity of C 65°C in the original report (Chen *et al.*, 2019 ▸) was higher than what we observed, indicating a significant experimental error for this specific procedure. Indeed, N_2_-sorption reports for COF-300 in the literature vary widely with a total N_2_ uptakes varying between almost zero up to 400 cm^3^ g^−1^ (Chen *et al.*, 2019 ▸; Fischbach *et al.*, 2019 ▸; Ma *et al.*, 2020 ▸). In contrast, the reproducibility of our developed method I 65°C was high with almost perfectly overlapping PXRD patterns and total N_2_ uptakes varying between 400 and 500 cm^3^ g^−1^ (Fig. S2 of the supporting information). Additionally, during our experiments, we noted that, to obtain optimal porosity, adequate sonication of the reaction mixture (as described in the supporting information) is of utmost importance.

**Figure 3:**
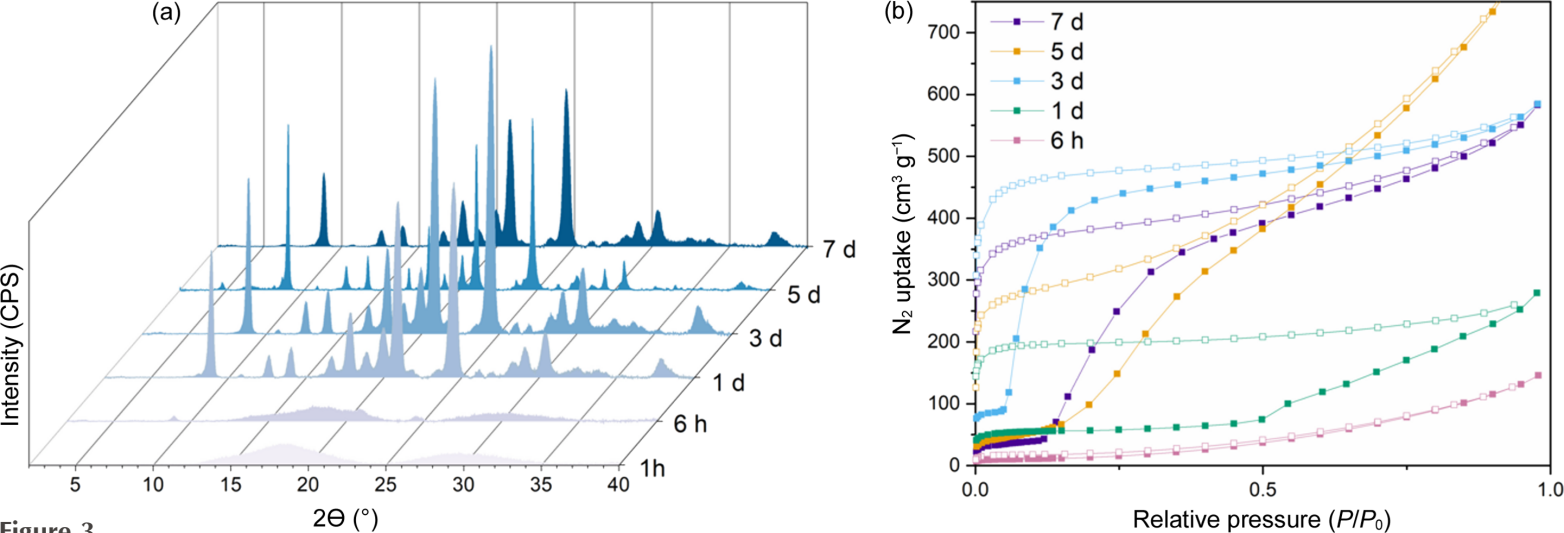
Influence of reaction time on I 65°C. (*a*) Background-corrected PXRD patterns of COF-300 treated at 65°C for 1 h, 6 h, 1 d, 3 d, 5 d and 7 d. (*b*) N_2_-sorption isotherms for COF-300 treated at 65°C for 6 h (pink), 1 d (green), 3 d (blue), 5 d (orange) and 7 d (purple).

In a next step, the influence of reaction time on the resulting materials was studied. For this, COFs were prepared for all four conditions (C RT, C 65°C, I RT and I 65°C) using six different reaction times: 1 h, 6 h, 1 d, 3 d, 5 d and 7 d. All samples obtained were analysed via PXRD and all crystalline materials were studied using N_2_-sorption. In Fig. 3 ▸, the results obtained for the I 65°C series are presented. Though the method employed with minor adaptations (*e.g.* THF as solvent) was recently reported to successfully synthesize fully developed COF-300 in 20 min (Wang *et al.*, 2023 ▸), it took 6 h for trace crystallinity to appear under the conditions used in this work. After 1 d, full crystallinity is obtained with negligible change when shifting to longer reaction times. However, a 7 d synthesis resulted in broader peaks, indicating slightly decreased crystallinity. Still, the effect of time on porosity is more pronounced with a clear maximum obtained after 3 d. Interestingly, samples treated for short times (6 h and 1 d) did not show the typical two-step COF-300 isotherm with a pore opening to N_2_ around 0.05 *P*/*P*_0_ (Chen *et al.*, 2019 ▸; Ma *et al.*, 2020 ▸). This potentially indicates an inability of the material to open up the pores due to incomplete building of the network. Alternatively, this could also indicate a stuffing of the inner, larger pores with building blocks and solvent molecules that are stuck. In contrast, synthesis times that are too long lead to a delayed and less sharp opening of the pores. This can be explained by defect formation in the inner structure, making the pores more irregular and larger. Indeed it is known that the TAM building block used can degrade in an acidic environment to form the dye pararosaniline (as shown in Scheme S2) (Gomberg, 1898 ▸), which is in agreement with the magenta colour observed in the reaction mixtures (which became more pronounced for longer synthesis times, as presented in Fig. S3). Additionally, Raman analysis showed the appearance of new peaks in a degraded sample compared with the fresh TAM sample as shown in Fig. S4. The new peaks could be matched to those reported as characteristic for pararosaniline (Kosanić & Tričković, 2002 ▸). As this degradation reaction essentially indicates the removal of one aniline molecule from the TAM structure, a shift to irregular and larger pores can be expected. Still, the number of degraded linkers should be limited, as the effect on the crystallinity is minor.

**Figure 4:**
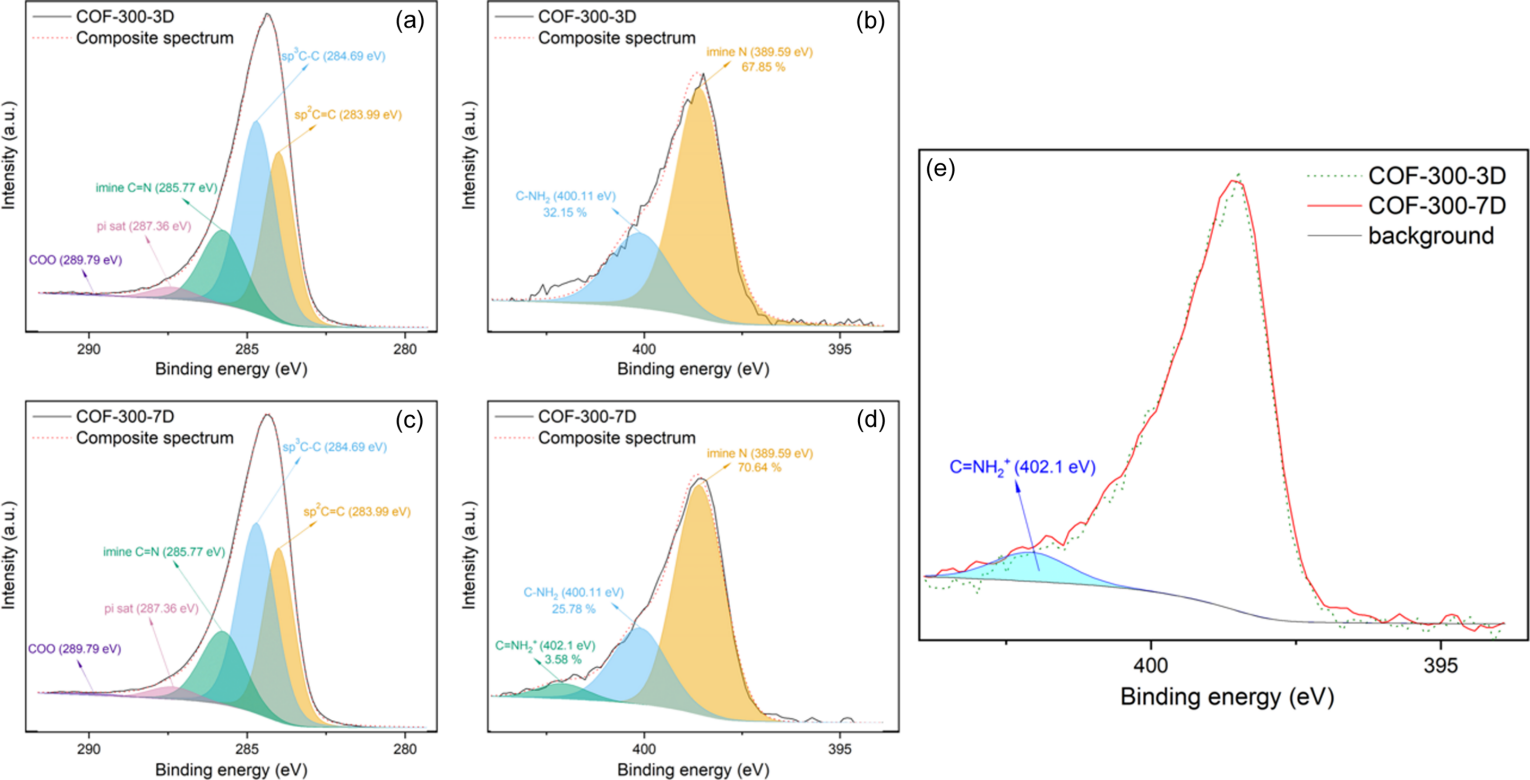
High-resolution XPS spectra for COF-300-3D: (*a*) C 1*s*, (*b*) N 1*s*; and for COF-300-7D: (*c*) C 1*s*, (*d*) N 1*s*. (*e*) Overlay of both N 1*s* spectra (COF-300-3D: dashed green; COF-300-7D: red) with the C=NH_2_^+^ contribution (cyan). The background is shown in black.

To confirm the presence of a degraded linker in the COF, high-resolution X-ray photoelectron spectroscopy (XPS) spectra were recorded for two samples: COF-300-3D synthesized according to the optimized principles (3 d reaction time at 65°C using Int) versus the defected material COF-300-7D (7 d reaction time at 65°C using Int). No significant differences between both samples could be observed in the C 1*s* spectra [Figs. 4 ▸(*a*) and 4 ▸(*c*)], with both spectra deconvoluting into five peaks with binding energies of 283.99, 284.69, 285.77, 287.36 and 289.79 eV. The first three peaks could be assigned to *sp*^3^ C (283.99 eV), *sp*^2^ C (284.69 eV) and imine C (285.77 eV) (Shehab *et al.*, 2021 ▸; Chaki Roy & Kundu, 2023 ▸). Additionally, the peak at 287.36 eV (labelled ‘π sat C’) could be interpreted as a satellite peak caused by energy loss by interaction with the aromatic electron cloud. Finally, the peak at 289.79 eV could be explained by the COO motif in the residual acetic acid in the sample (Mudiyanselage *et al.*, 2019 ▸). Note that the contribution of this peak was almost insignificant (<1%). The N 1*s* spectrum for COF-300-3D [Fig. 4 ▸(*b*)] could be deconvoluted into contributions of imine N (389.59 eV, 67.85%) and free amine groups (400.11 eV, 32.15%) (Bai *et al.*, 2022 ▸). As the synthesis technique employed makes use of an imine-exchange procedure yielding an imine as well as an amine (Fig. 1 ▸), the presence of free amine groups in the resulting material is to be expected. Those free amine groups could be present as both residual aniline stuck on the material or unreacted amine moieties in the TAM linker. However, given the excellent porosity and crystallinity of COF-300-3D, it could be concluded that the influence on the structure is minimal. Finally, the N 1*s* spectrum for COF-300-7D is presented in Fig. 4 ▸(*d*). For this sample, aside from the previously mentioned imine N (70.64%) and amine N (25.78%), a third contribution could be observed during deconvolution, namely C=NH_2_^+^ (402.1 eV) (Bai *et al.*, 2022 ▸). This could be assigned to the degradation of TAM, which is characterized by the formation of a C=NH_2_^+^ moiety as shown in Scheme S2. Though its presence is limited (3.58%), comparison of the N 1*s* spectra for both samples [Fig. 4 ▸(*e*)] indeed shows a clear shoulder around 402 eV for COF-300-7D compared with COF-300-3D. Interestingly, the amount of imine N observed has increased in this sample compared with COF-300-3D, indicating that even though some degradation occurs, the error-correction process is still efficient. As the TAM linker is used in the majority of 3D COFs (Guan *et al.*, 2020 ▸; Bourda *et al.*, 2021 ▸), this degradation process in a slightly acidic environment can have a significant impact on the quality of such materials and sufficient knowledge about this is of utmost importance.

In Figs. S6–S8, the effect of time on I 65°C (as discussed earlier) is compared with the effect on the other samples. Note that, as expected, I RT is slower to form a crystalline material compared with I 65°C due to the reduced error correction at room temperature, with reflections appearing after 1 d, and fully developed crystallinity after 5 d. However, the pore structure never fully establishes, as indicated by the broad, late and small second step in the N_2_-sorption isotherm. The appearance of crystallinity in C 65°C is even more delayed, with no crystalline reflections observed after 1 d of reaction time, indicating the superiority of the intermediate-assisted procedure. Here, maximal crystallinity is observed after 5 d, as peaks start to broaden significantly after 7 d. Surprisingly, the best N_2_-sorption behaviour was observed for the 7 d sample, indicating that the relationship between crystallinity and porosity is not always straightforward. Finally, using the conditions of C RT, we were unable to form any crystalline material, even after 7 d of reaction time. We also checked if the scale of the synthesis had any influence on the material. Therefore, a sample (I 65°C ×5) was prepared in an identical way to I 65°C but with every quantity used multiplied by 5. The resulting PXRD patterns and the N_2_-sorption isotherms are presented in Fig. S9 and show no significant influence on the crystallinity and a small decrease of porosity (with a BET surface area of 1180 m^2^ g^−1^ and *V*_p_ of 0.71 obtained for I 65°C ×5).

**Figure 5:**
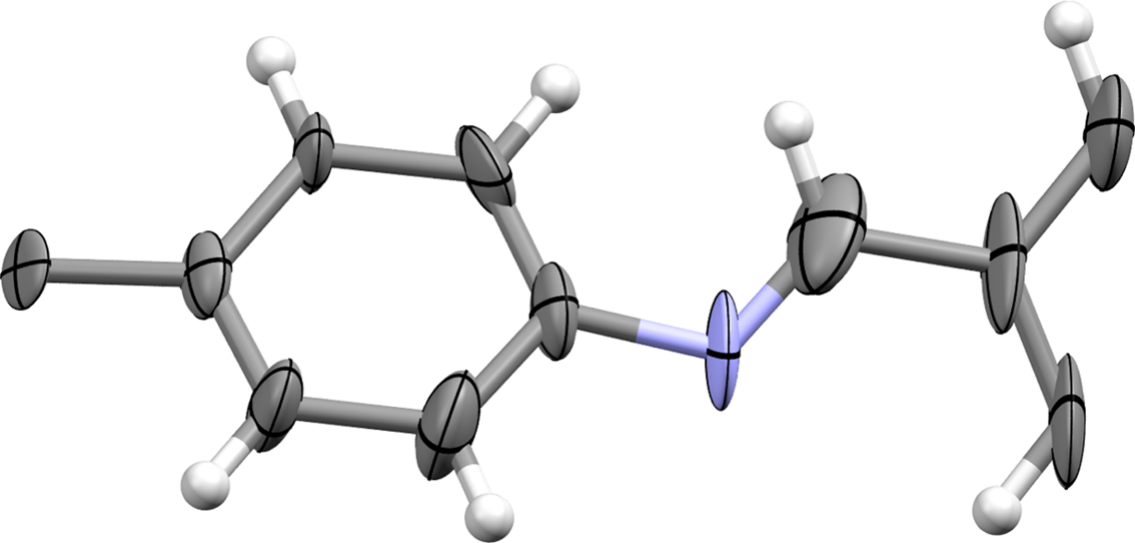
Asymmetric unit of COF-300 as determined by 3DED. Thermal displacement ellipsoids are shown at the 50% probability level.

**Figure 6:**
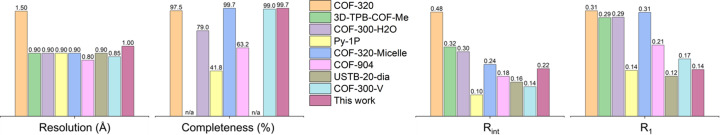
Comparison of data collection statistics and final *R* values obtained for selected reports of full COF structure solution via 3DED: COF-320 (Zhang *et al.*, 2013 ▸), 3D-TPB-COF-Me (Gao *et al.*, 2019 ▸), COF-300-H2O (Sun *et al.*, 2019 ▸), py-1P (Kang *et al.*, 2022 ▸), COF-320-micelle (Zhou *et al.*, 2023 ▸), COF-904 (Xiao *et al.*, 2023 ▸), USTB-20-dia (Yu *et al.*, 2023*a* ▸), COF-300-V (Sun *et al.*, 2023 ▸), COF-300 (this work). A full comparison for COFs where 3DED could locate all non-hydrogen atoms is given in Table S3.

After assessing the best conditions for the synthesis of COF-300, the optimized sample (I 65°C) was used for further analysis. SEM imaging (Fig. S10) showed clear nanocrystallinity with a particle size around 600 nm, ideal for 3DED analysis. For this, a Rigaku XtaLAB Synergy-ED operated at room temperature with an electron wavelength of 0.0251 Å was used. Within 10 min, datasets for two separate crystals could be collected, showcasing high resolution (0.80 Å), low *R* values (≤0.2) and high completeness (≥90%). Note that a resolution cut-off was applied at 1 Å as the 〈*F*^2^/σ*F*^2^〉 value started to drop significantly (close to 1). Even improved statistics could be obtained by merging of the two datasets. Full statistics, grain snapshots and diffraction images of the crystals used can be found in Tables S1 and S2 and Fig. S11 of the supporting information. The datasets obtained were processed using *CrysAlisPro* and kinematically refined using *SHELXL* (Sheldrick, 2015*a* ▸) within the graphical interface of *Olex2* (Dolomanov *et al.*, 2009 ▸) using RIGU restraints. All non-hydrogen atoms could be located during initial structure solution via *SHELXT* (Sheldrick, 2015*b* ▸). Although individual twin domains could not be observed in images taken from these phases (Tomioka *et al.*, 2002 ▸), during refinement twinning by merohedry (twin law: 0 1 0 1 0 0 0 0 −1, twin fraction: 12%) was encountered and further applied as such. The same twin law could also be observed in the unmerged datasets with domain percentages of 33 and 6%, respectively. Note that this twin law corresponds to twinning around the twofold axis along [110], which is a symmetry element of the higher symmetry point group 4/*mmm* compared with 4/*m*. This type of merohedral twinning is often observed in low-symmetry tetragonal space groups such as *I*4_1_/*a* (Parsons, 2003 ▸). For the final, merged dataset (*R*_int_ 22.04%), the asymmetric unit of the refined structure is shown in Fig. 5 ▸ (*R*_1_ = 13.72%) and a packing diagram is presented in Fig. S12. In Fig. 6 ▸, the statistics obtained are compared with a representative selection of COFs where 3DED allowed us to locate all non-hydrogen atoms during structure solution (a complete comparison can be found in Table S3). From this it could be noted that the data quality obtained is often still too poor for accurate structure determination with low resolution, low completeness or high *R* values. It appears that some studies even fail to report essential parameters as data completeness. Additionally, it is clear that some recent studies show better *R* values compared with our data. This could be explained by increased beam damage for our room-temperature measurements compared with the low temperatures used in the literature (Gruene & Mugnaioli, 2021 ▸; Huang *et al.*, 2021*c* ▸).

**Figure 7:**
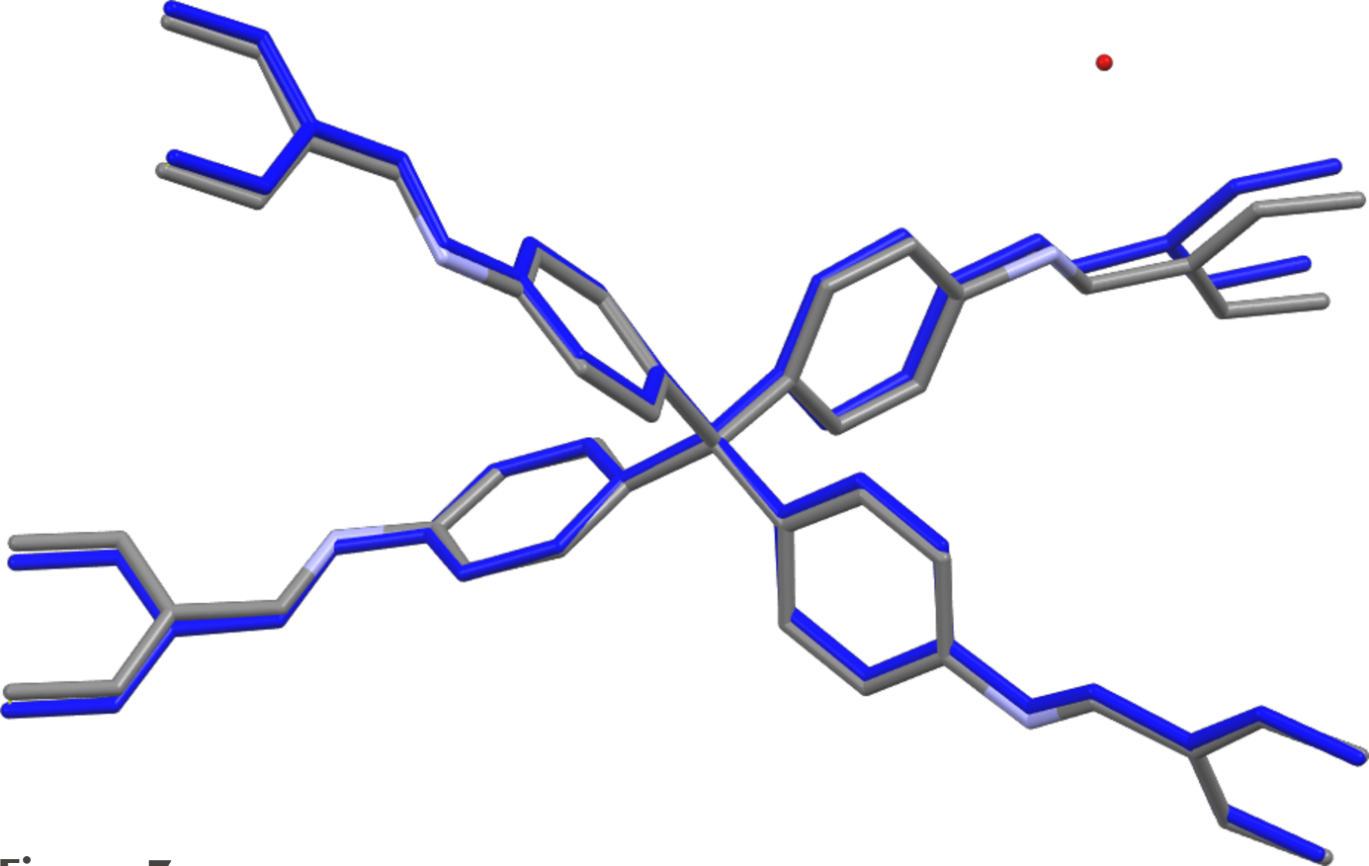
Fit of the COF-300 structure as obtained by synchotron SCXRD (Ma *et al.*, 2018*b* ▸) (COF-300_hydrated, grey) and 3DED (COF-300, blue) using a capped stick model. Hydrogens are omitted for clarity. Obtained RMSD of 0.234.

To confirm the validity of the 3DED structure analysis, the structure obtained was compared with the synchotron radiation SCXRD structure solution published by Ma *et al.* (2018*b* ▸). An overlay of both structures (Fig. 7 ▸) shows negligible differences with a root-mean-square deviation (RMSD) of 0.234. The main difference between both structures is associated with the larger unit cell (*a* = *b* = 20.7 Å, *c* = 8.8 Å) and subsequently increased volume for the 3DED structure (Table 1 ▸) compared with the SCXRD structure (versus *a* = *b* = 19.6 Å, *c* = 8.9 Å). However, this can be readily attributed to the difference in measurement temperature (293 versus 100 K for the 3DED structure and the SCXRD structure, respectively). Additionally, because of the lower resolution and higher measurement temperature of the 3DED data, the position of the H_2_O molecules in the pores could not be resolved. However, when applying a solvent mask to our data, the presence of 40 electrons in the void was observed, consistent with the presence of four H_2_O molecules in the pore per unit cell. The lower number of H_2_O molecules observed compared with the SCXRD report might be caused by the ultra-high vacuum applied for 3DED measurements. Interestingly, a recent report showed that 3DED data recorded at high resolution (<0.8 Å) and cryo temperature (100 K) could resolve the position of the guest solvent molecules in the pores of COF-300 (Sun *et al.*, 2023 ▸).

**Figure 8:**
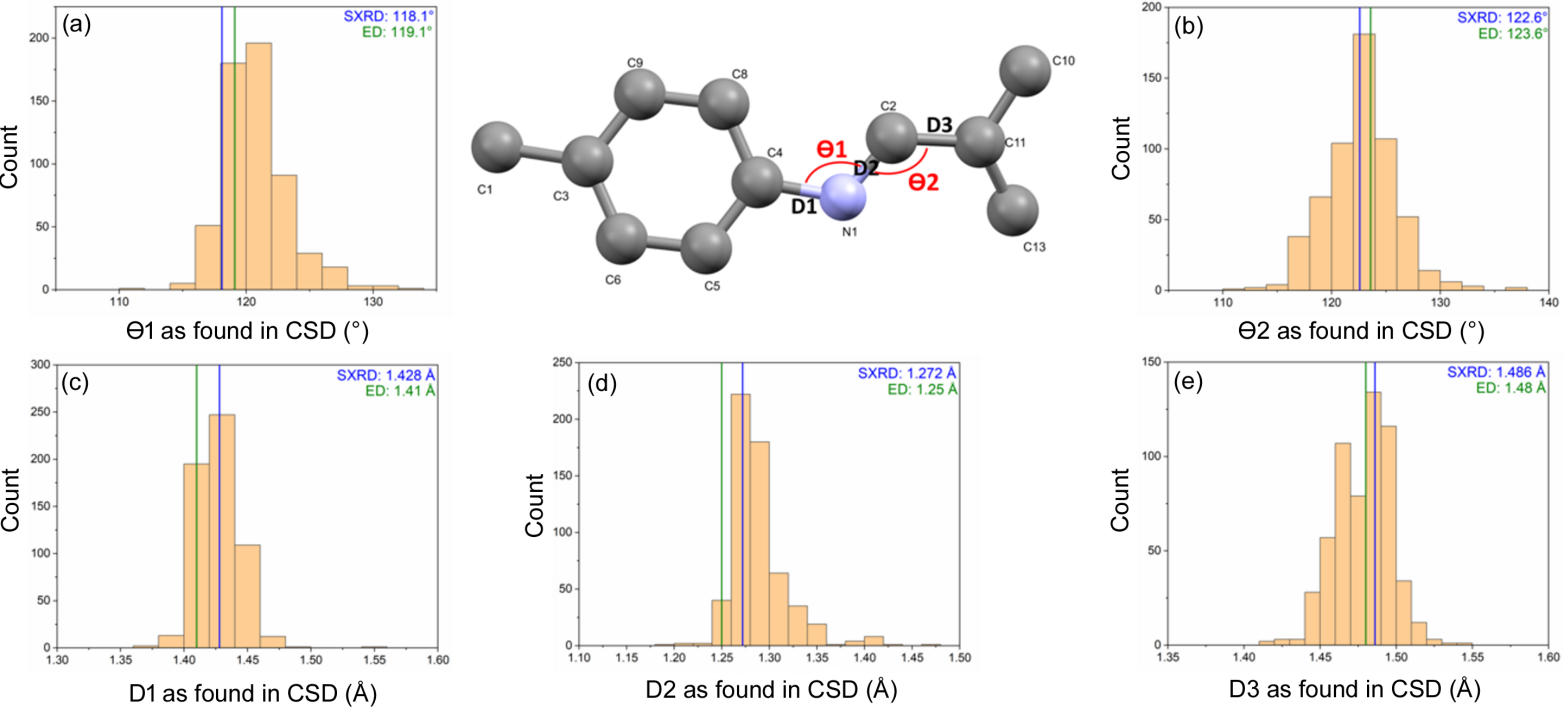
Structural details of the imine connection as encountered in COF-300 (phenyl–N=CH–phenyl). SCXRD data [COF-300_hydrated, blue (Ma *et al.*, 2018*b* ▸)] and 3DED data (COF-300, green) are compared with values reported in the CSD (Groom *et al.*, 2016 ▸). A numerical comparison, including standard deviations, can be found in Table S6. Observed bonds and angles are illustrated on the central drawing of the asymmetric unit: (*a*) Θ1, (*b*) Θ2, (*c*) D1, (*d*) D2, (*e*) D3.

As a final check for the accuracy of the obtained structure, the bond lengths and angles were compared with those observed for the SCXRD structure model (Ma *et al.*, 2018*b* ▸). Additionally, for the most vital part of the structure, the imine bond connecting the two linkers, mean bond lengths and angles were calculated from 438 related structures found in the CSD (Groom *et al.*, 2016 ▸) (resulting in 580 different imine bonds). The results are presented in Fig. 8 ▸ and Tables S4–S6. It can be seen from these data that both models are relatively close to each other, with the main differences found in the phenyl rings. Deviations from the ideal 120° bond angle observed for aromatic rings were slightly increased for the 3DED structure compared with the SCXRD structure, especially around the atoms bonded to a substituent (C3, C4 and C11). For example, the aromatic C3—C6—C5 angle increased from 120.4° (4) for the SCXRD structure to 122.4° (10) for the 3DED structure, as presented in Table S5. When comparing the imine bonds in both COF structures to the bond lengths and angles obtained in our CSD search, it could be concluded that both models show realistic values, within one standard deviation from the mean values found. From this it can be concluded that it is indeed possible to generate an accurate structure model from the 3DED data. Still, there is room for growth, as the presently used room-temperature measurements are unable to locate the position of guest atoms in the pores (Sun *et al.*, 2023 ▸) and might cause degradation of the COF structure, limiting the achievable resolution (Sun *et al.*, 2019 ▸). Furthermore, application of dynamical refinement protocols (Palatinus *et al.*, 2015 ▸) might greatly reduce the final *R* values (Sun *et al.*, 2023 ▸) as well as increase the precision of the determination of bond lengths. For example, while the value obtained here for D2 [1.25 Å, Fig. 8 ▸(*d*)] is on the low side compared with values found in the CSD [mean: 1.288 Å (30)], the high standard deviation (2) for D2 makes the difference almost insignificant. However, an accurate dynamically refined structure could not be modelled using the merged data in this work, which could be attributed to the combination of the observed twinning and low 〈*F*^2^/σ*F*^2^〉 values (Table S2).

## 3. Conclusions

The response of COF-300 to an intermediate-assisted synthesis protocol was studied by careful evaluation of the evolution of both crystallinity and porosity as functions of reaction time and temperature. Kinetic studies among four different synthesis conditions revealed three distinct stages in the synthesis of COF-300, namely a network build-up phase at short synthetic times (≤1 d) with low crystallinity and no pore flexibility, followed by an optimal stage (3 d) characterized by high crystallinity and porosity before partial breakdown by TAM degradation (≥5 d). This degradation process could be confirmed in both control experiments as well as the obtained COF materials and can easily be estimated by the observation of magenta-coloured reaction mixtures. As a pronounced influence of this degradation reaction on both crystallinity and porosity was observed and most 3D COFs are based on the TAM linker, knowledge of TAM degradation in a acidic environment is of utmost importance for the synthesis of high-quality 3D COFs. Knowledge of this degradation process might help to increase the synthetic toolbox for 3D COFs (which are mainly based on the TAM linker), which is still lacking compared with 2D COFs. However, using the optimized conditions, a reliable crystal structure of COF-300 could be readily obtained via 3DED analysis, indicating single crystallinity of the synthesized materials. The structure model obtained showed high completeness and comparable resolution and *R* values. Comparison with an SCXRD structure model as well as with data for similar chemical functionalities in the CSD database showed no significant differences, supporting that 3DED is a reliable and fast technique for the structure solution of COFs. As SCXRD structure solution is hardly possible and PXRD models often show ambiguity in structure determination, 3DED might play an important role in the future of COFs with better accessibility of 3DED diffraction equipment and improving dynamic refinement algorithms.

## 4. Related literature

The following references are cited in the supporting information: Rigaku Oxford Diffraction (2022 ▸); Kang *et al.* (2023 ▸); Cheng *et al.*(2023 ▸); Bruno *et al.* (2002 ▸).

## Supplementary Material

Crystal structure: contains datablock(s) 1. DOI: 10.1107/S2052252524003713/vq5005sup1.cif

Structure factors: contains datablock(s) 1. DOI: 10.1107/S2052252524003713/vq5005sup2.hkl

Supporting Information - revised - highlighted. DOI: 10.1107/S2052252524003713/vq5005sup3.pdf

CCDC reference: 2321626

## Figures and Tables

**Table 1:** Crystal data and structure refinement statistics for COF-300 analysed via SCXRD (Ma *et al.*, 2018*b* ▸) and 3DED (this work)

	SCXRD (COF-300_hydrated)	3DED (COF-300)
Merged datasets	1	2
Empirical formula	C_82_H_72_N_8_O_8_	C_41_H_28_N_4_·H_2_O
Resolution (Å)	0.83	1.00
Formula weight	1297.47	594.72
Temperature (K)	100	298 (1)
Crystal system	Tetragonal	Tetragonal
Space group	*I*4_1_/*a*	*I*4_1_/*a*
*a* (Å)	19.6394 (9)	20.7 (3)
*b* (Å)	19.6394 (9)	20.7 (3)
*c* (Å)	8.9062 (4)	8.8 (2)
α (°)	90	90
β (°)	90	90
γ (°)	90	90
*V* (Å^3^)	3435.2 (4)	3755 (157)
*Z*	2	4
ρ_calc_ (g cm^−3^)	1.254	1.052
*F*(000)	1368.0	507.0
Crystal size (mm)	0.06 × 0.01 × 0.01	0.0006
Radiation	Synchrotron (λ = 1.0332)	TEM (λ = 0.0251)
2θ range (°)	6.032–77.016	0.178–1.438
Index ranges	−22 ≤ *h* ≤ 23, −17 ≤ *k* ≤ 23, −10 ≤ *l* ≤ 10	−20 ≤ *h* ≤ 20, −20 ≤ *k* ≤ 20, −8 ≤ *l* ≤ 8
Reflections collected	9358	7656
Independent reflections	1569 (*R*_int_ = 0.0687, *R*_sigma_ = 0.0599)	983 (*R*_int_ = 0.2204, *R*_sigma_ = 0.0916)
Data/restraints/parameters	1569/0/114	983/81/103
Goodness-of-fit on *F*^2^	1.101	1.316
Final *R* indexes [*I* ≥ 2σ (*I*)]	*R*_1_ = 0.0903, ω*R*_2_ = 0.2323	*R*_1_ = 0.1372, ω*R*_2_ = 0.3425
Final *R* indexes (all data)	*R*_1_ = 0.1424, ω*R*_2_ = 0.2617	*R*_1_ = 0.1808, ω*R*_2_ = 0.3793
Largest difference peak/hole (e Å^−3^)	0.29/−0.35	0.14/−0.15
Additional information	Data taken from Ma *et al.* (2018*b* ▸)	Merohedral twin: (0 1 0 1 0 0 0 0 −1), twin fraction: 12%
