# Supplementary material for: Analysis of COF-300 synthesis: probing degradation processes and 3D electron diffraction structure
Source: IUCrJ. 2024 May 10;11(Pt 4):510–8. doi: 10.1107/S2052252524003713 (PMC11220877; doi:10.1107/S2052252524003713)
Supplement: Supplementary file 3 [file m-11-00510-sup3.pdf]

IUCrJ

Volume 11 (2024)

Supporting information for article:

Analysis of COF-300 Synthesis: Probing Degradation Processes and 3D Electron Diffraction Structure.

Laurens, Subhrajyoti, Sho, Christian R., Pascal, Kristof, Laurens, Subhrajyoti, Sho, Christian R., Pascal and Kristof

<u>**Supporting Information**</u>

Analysis of COF-300 Synthesis: Probing Degradation Processes and 3D Electron Diffraction Structure

Laurens Bourda, Subhrajyoti Bhandary, Sho Ito, Christian R. Göb, Pascal Van Der Voort and Kristof Van Hecke*

Section S1. General procedures

Materials

Unless stated otherwise all reagents were purchased from commercial sources and used without further purification.

Instrumentation

The chemical structure of the materials was analyzed using 1H NMR in $CDCl_3$ on a Bruker 300 MHz AVANCE spectrometer with chemical shifts (d) expressed in ppm relative to a tetramethylsilane standard. The ^{13}C CP-MAS solid-state nuclear magnetic resonance (NMR) spectra were recorded at ambient temperature on a 500MHz Bruker Avance III spectrometer using a dual channel CPMAS iProbe - 1H and Broadband with 4 mm zirconia rotors. The temperature was set to 298 K and controlled by the Bruker BVT controller throughout the measurements. The magic adjustment is done automatically using the iProbe functionality and a 1D ^{79}Br measurement on a KBr sample at a spinning frequency of 5kHz. Here the number of spinning sidebands is maximized in both numbers and amplitude in order to achieve the optimal magic angle value (± 54.74°). Following magic angle adjustment, the probe was shimmed on the ^{13}C signals from an adamantane reference sample where the shims were adjusted until the peak width at half height of the two signals in question was between 2 – 5 Hz. The Cross-polarization with MAS (CP-MAS) measurements were used to acquire the ^{13}C data at 125.770 MHz. The 1H ninety-degree pulse width was set to 2.5 µs. The CP contact time was set to 2 ms. High power swept-frequency two-pulse phase modulation (swftppm) ^{1}H decoupling was applied during data acquisition (40 ms). The decoupling frequency was set to 2900 Hz, on resonance with the main 1D ^{1}H signal. Finally, the MAS sample-spinning rate was 12 kHz and the relaxation delay between scans was set to 5 seconds. The ^{13}C chemical shifts are given relative to the methylene carbon signal of adamantane assigned to 37.77 ppm as secondary reference. Nitrogen adsorption-desorption isotherms were obtained using a micromeretics Tristar II measured at 77 K. Powder X-ray diffraction (PXRD) patterns were collected on a Bruker D8 Advance diffractometer equipped with an autochanger and LynxEye XE-T Silicon strip Line detector, operated at 40 kV, 30 mA using Cu-Kα radiation (λ = 1.5406 Å) in Bragg-Brentano geometry. Scanning electron microscopy (SEM) measurements were performed using a FEI Quanta 200 FSEM. The surface chemical composition of the COFs was analyzed via XPS measurements. To do so, a PHI 5000 Versaprobe II spectrometer (ULVAC-Physical Electronics) equipped with a monochromatic Al Kα X-ray source (hν = 1486.6 eV) and operating at a power of 25 W (beam size of 100 µm) was employed. The pressure of the main XPS chamber was constantly kept below 10−6 Pa during the measurements. The emitted photoelectrons were detected with a hemispherical analyzer placed at an angle of 45° relative to the plane of the samples. Survey scans and high-resolution C1s and N1s spectra were recorded at pass energies of 187.85 eV (0.8 eV step size) and 23.5 eV (0.1 eV step size), respectively. The acquired survey scans (0–1100 eV) were then analyzed via Multipak software (version 9.6) to determine and quantify the present surface elements after applying a Shirley background subtraction with the relative sensitivity factors provided by the manufacturer of the instrument. Electron Diffraction (3D ED) data were recorded at room temperature using a wavelength of 0.0251 Å on a Rigaku XtaLAB Synergy-ED equipped with a JEOL 200 kV LaB_6 electron source and a Rigaku HyPix-ED detector. The images were

interpreted and integrated with the program CrysAlisPro.[1] Using Olex2,[2] the structures were solved by intrinsic phasing using the ShelXT structure solution program and refined by full-matrix least-squares on F^2 using the ShelXL program package.[3,4] Non-hydrogen atoms were anisotropically refined and the hydrogen atoms in the riding mode with isotropic temperature factors fixed at 1.2 times U(eq.) of the parent atoms. A solvent mask was calculated and 40 electrons were found in a volume of 1056 $Å^3$ in 1 void per unit cell. This is consistent with the presence of $4[H_2O]$ per Unit Cell which account for 40 electrons per unit cell.

Section S2: Synthesis

Synthetic procedure for Intermediate (Int)

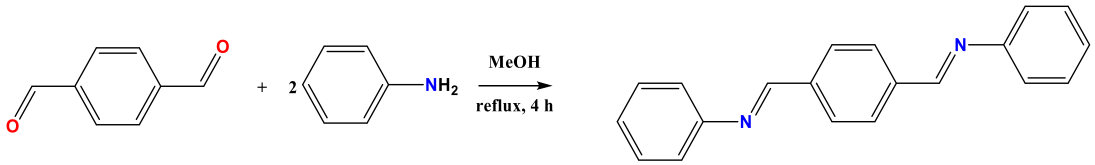

Scheme S1: Synthesis of(1E,1'E)-1,1'-(1,4-phenylene)bis(N-phenylmethanimine)

4.47 mL aniline and 3.53 g terephtalaldehyde (TA) were dissolved in 100 mL methanol. The resulting solution was refluxed (80 °C) for 4 h and filtered. The product was purified by stirring in fresh dry MeOH overnight and a second filtration to obtain pure (1E,1'E)-1,1'-(1,4-phenylene)bis(N-phenylmethanimine) (Intermediate, Int). Single crystals of the product were obtained by slow addition of hexane to a dichloromethane solution of Int.

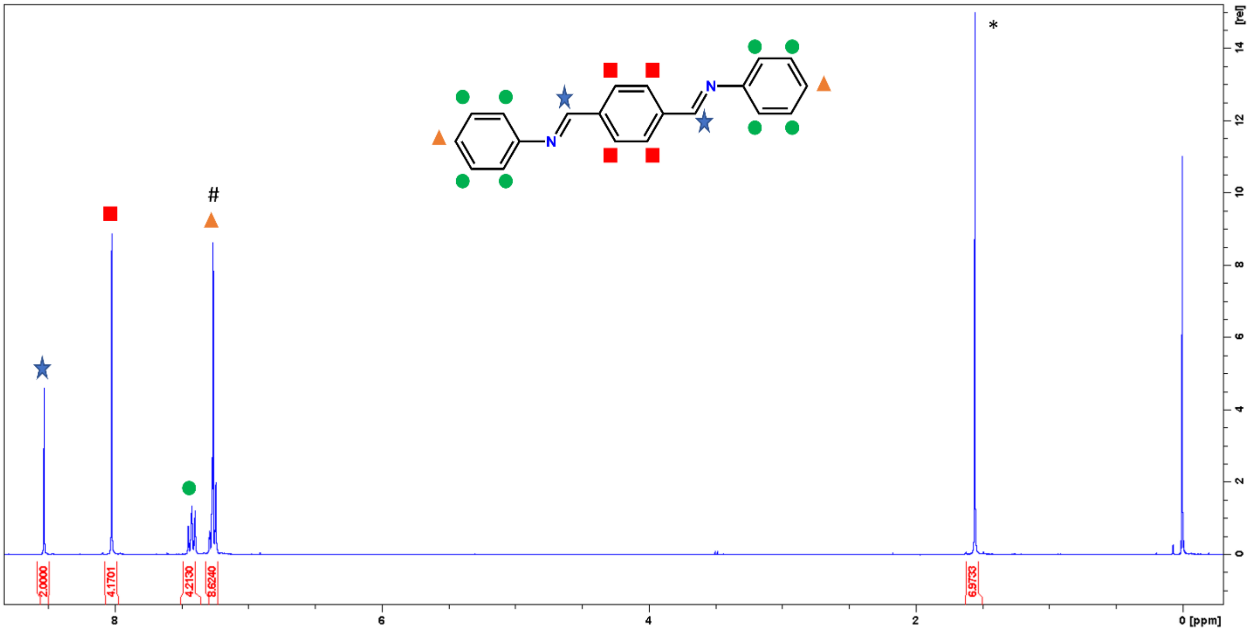

Figure S1: NMR spectra of Int, the solvent ($CDCl_3$) peak is depicted with a #, the peak marked with a * was assigned to residual H_2O in the sample.

Synthetic procedure for COF-300 (I 65°C)

30 mg Int and 20 mg tetra-(4-anilyl)-methane (TAM) were mixed in 1.6 mL 1,4-dioxane and 0.4 mL cyclohexane and treated in an ultrasound bath for 5 minutes. Subsequently, 0.4 mL 3 $molL^{-1}$ acetic acid was added, and the mixture was placed in an oven at 65 °C for 72 hours. The COF was obtained after filtration, washing with 1,4-dioxane and diethylether and drying on the filter.

For samples of the C series 12 mg terephthalaldehyde (BDA) was used instead of 30 mg Int.
Samples of the RT series were simply left on the lab bench for 72 hours instead of being placed in an oven.
For the time studies reaction times in the oven were varied from 1 hour (1 h) over 6 hours (6 h), 24 hours (1 d), 72 hours (3 d), 120 hours (5 d) to 168 hours (7 d).

Degradation test for TAM

To test the degradation of TAM a COF synthesis mixture was made but without addition of the aldehyde/intermediate: 20 mg (TAM) was dissolved in 1.6 mL 1,4-dioxane, 0.4 mL cyclohexane and 0.4 mL 3 $molL^{-1}$ acetic acid and treated in the ultrasound bath until a clear solution was obtained. The sample was then treated at 65 °C for 7 days to obtain degraded TAM as visible by the pink color of the solution. The (colorless) organic layer was subsequently decanted before further analysis was performed on the aqueous layer.

The same procedure was followed for the fresh TAM sample except for the treatment of 65 °C. Here, the organic layer was decanted immediately after dissolving TAM and further analysis was performed as fast as possible to avoid degradation.

Section S3: Characterization

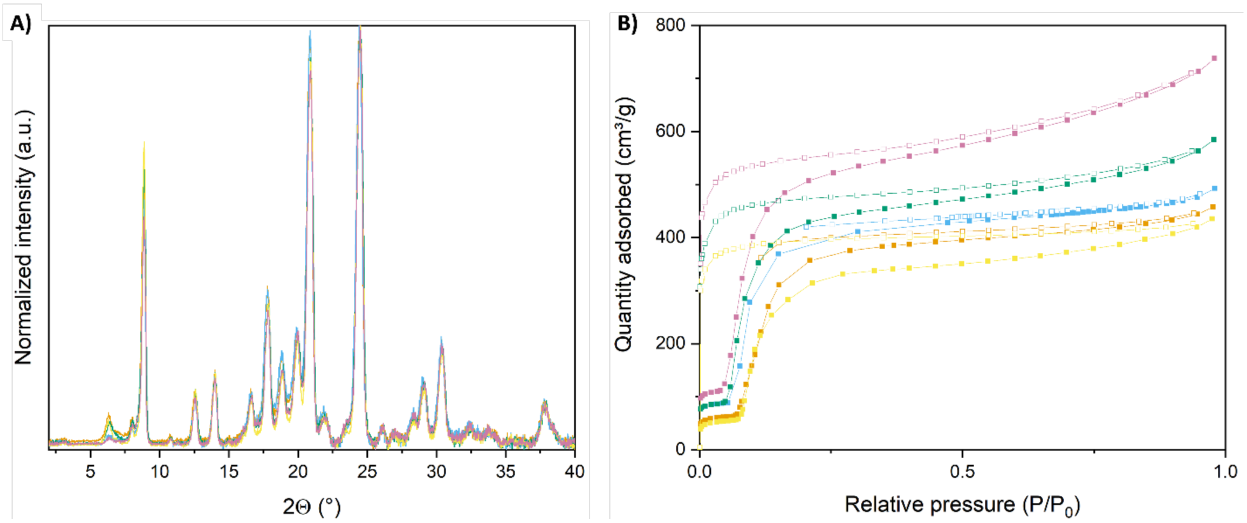

Figure S2: A) Normalized, background corrected PXRD patterns and B) N_2-sorption isotherms of different batches of I 65°C.

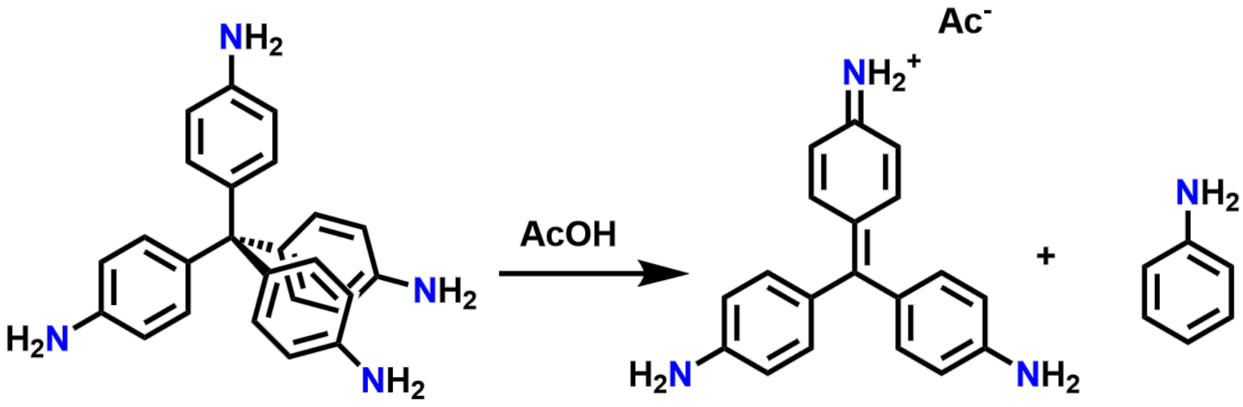

Scheme S2: Proposed degradation reaction from TAM to pararosaniline.

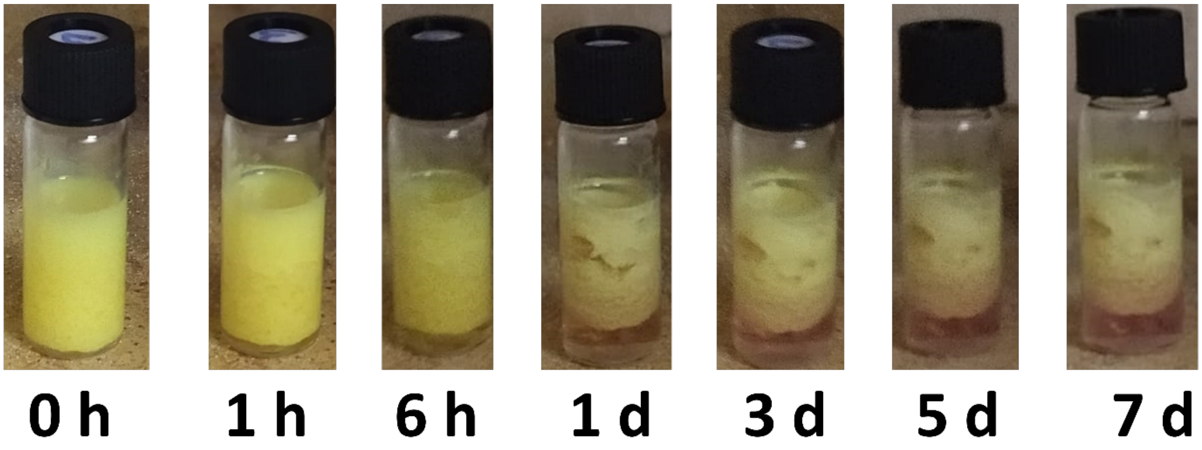

Figure S3: Visual evolution of the COF synthesis (I 65°C) over time. Magenta discoloration in the aquatic (bottom) layer is slowly increasing over time.

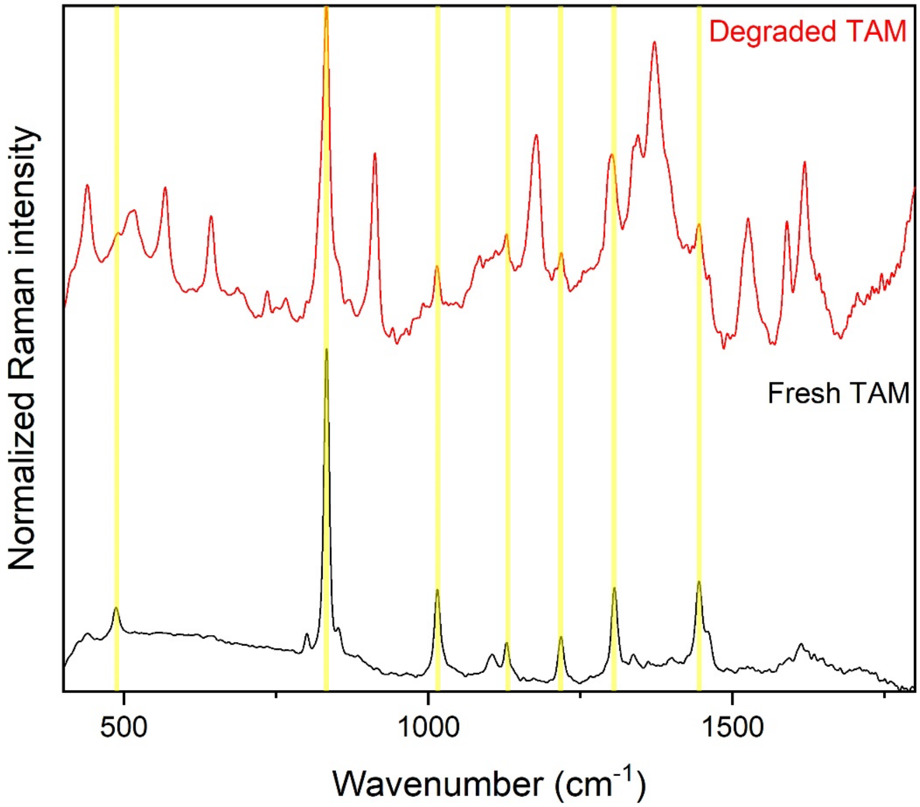

Figure S4: Normalized Raman spectra of fresh TAM aqueous solution (black) and degraded TAM aqueous solution (red).

A)

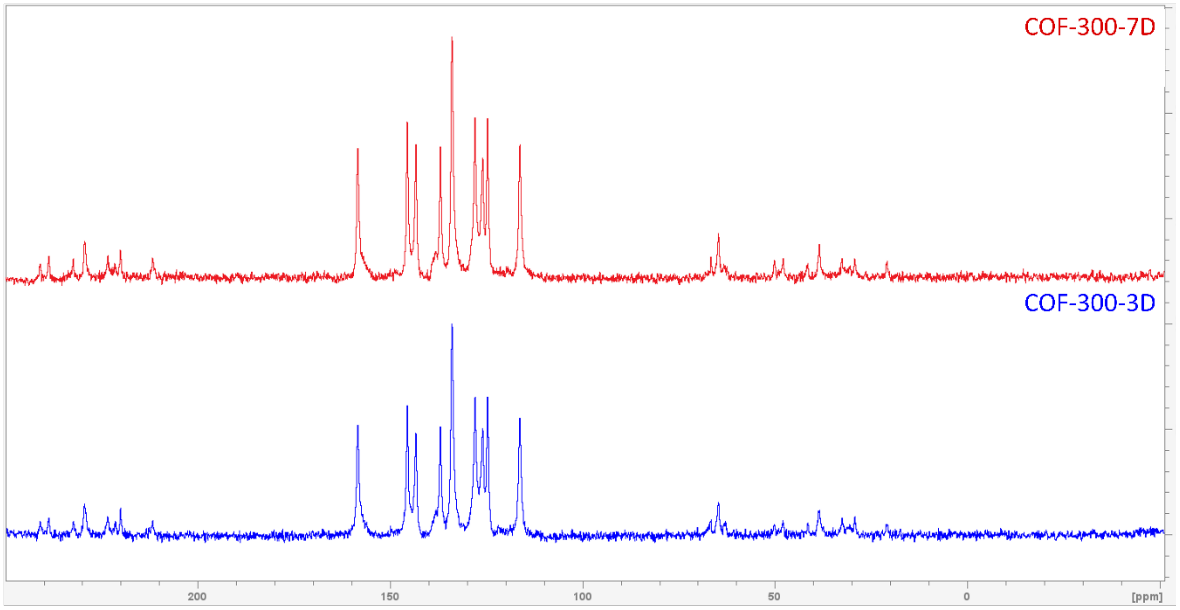

B)

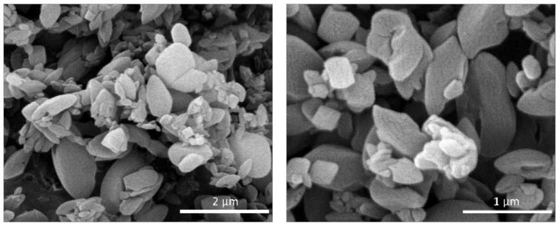

COF-300-3D

C)

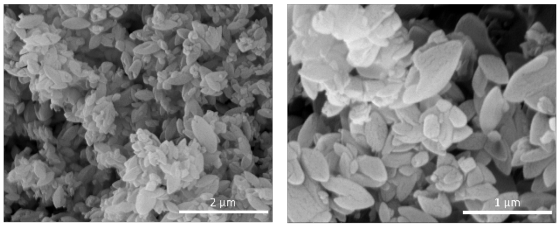

COF-300-7D

Figure S5: A) Solid-state ^{13}C CP-MAS spectrum of COF-300-3D (blue) and COF-300-7D (red); B) SEM images for COF-300-3D; C) SEM images for COF-300-7D.

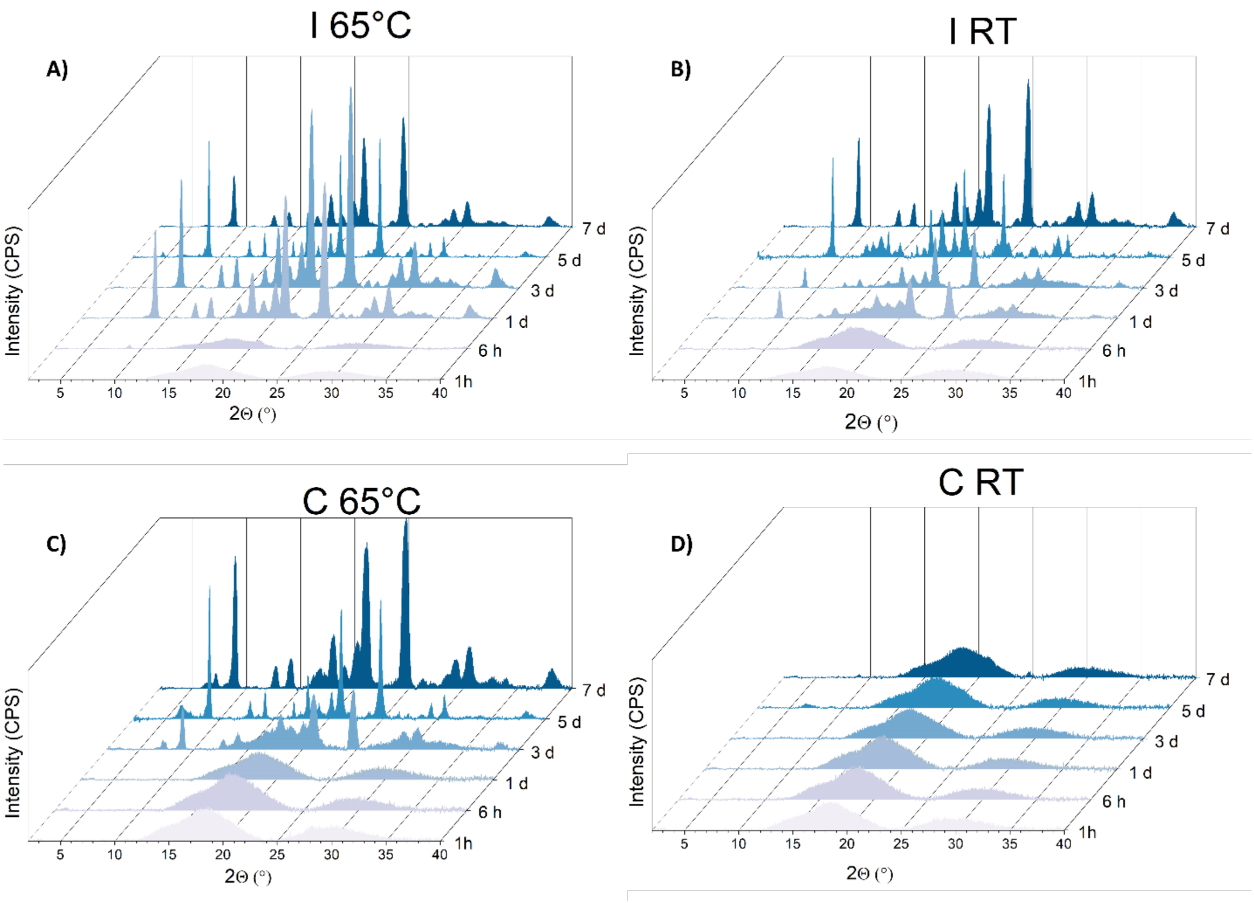

Figure S6: Background corrected PXRD patterns for COF-300 synthesized using different reaction times: 1 h, 6 h, 1 d, 3 d, 5d and 7 d. Conditions used were: A) I 65 °C; B) I RT; C) C 65 °C; D) C RT.

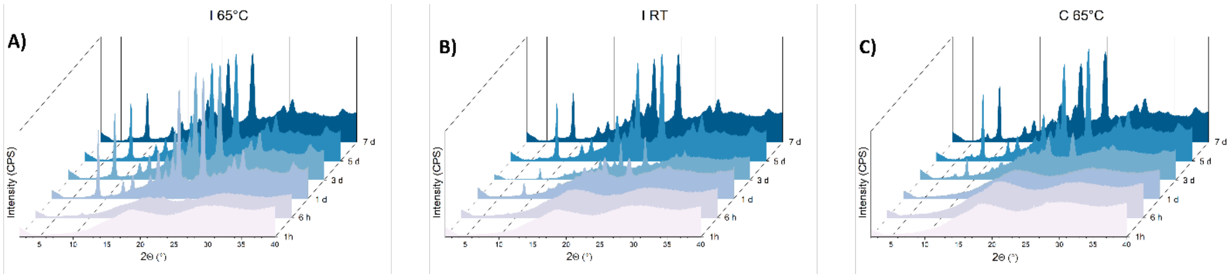

==**Figure S7:** Uncorrected PXRD patterns for COF-300 synthesized using different reaction times: 1 h, 6 h, 1 d, 3 d, 5d and 7 d. Conditions used were: A) I 65 °C; B) I RT; C) C 65 °C. Broad peaks around 18° and 30° could be assigned to the use of typical polymeric holders.==

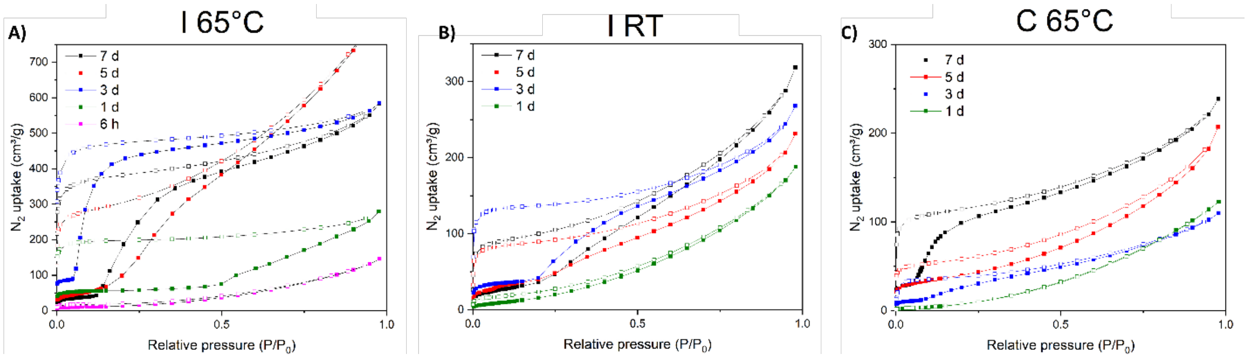

Figure S8: N_2-sorption isotherms for COF-300 synthesized using different reaction times: 6 h (pink), 1 d (green), 3 d (blue), 5d (red) and 7 d (black). Conditions used were: A) I 65 °C; B) I RT; C) C 65 °C.

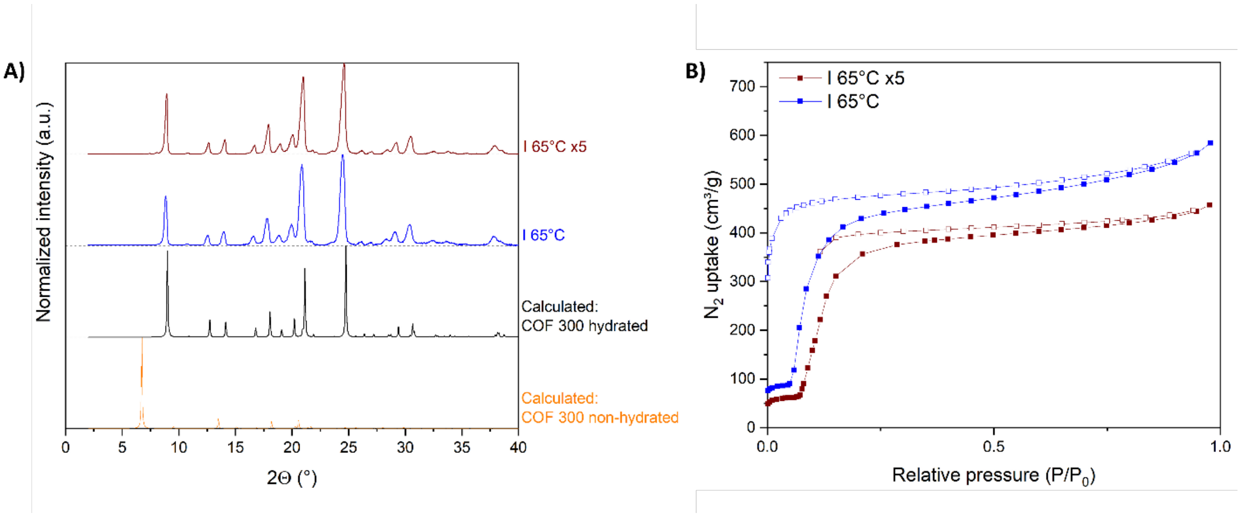

Figure S9: Normalized background corrected PXRD and N2-sorption analysis of I 65 °C (blue) compared to its upscaled version (I 65 °C x5, brown). Calculated patterns, based on the single-crystal structure of COF-300 (orange) and COF-300 hydrated (black)[5] are included for clarity.

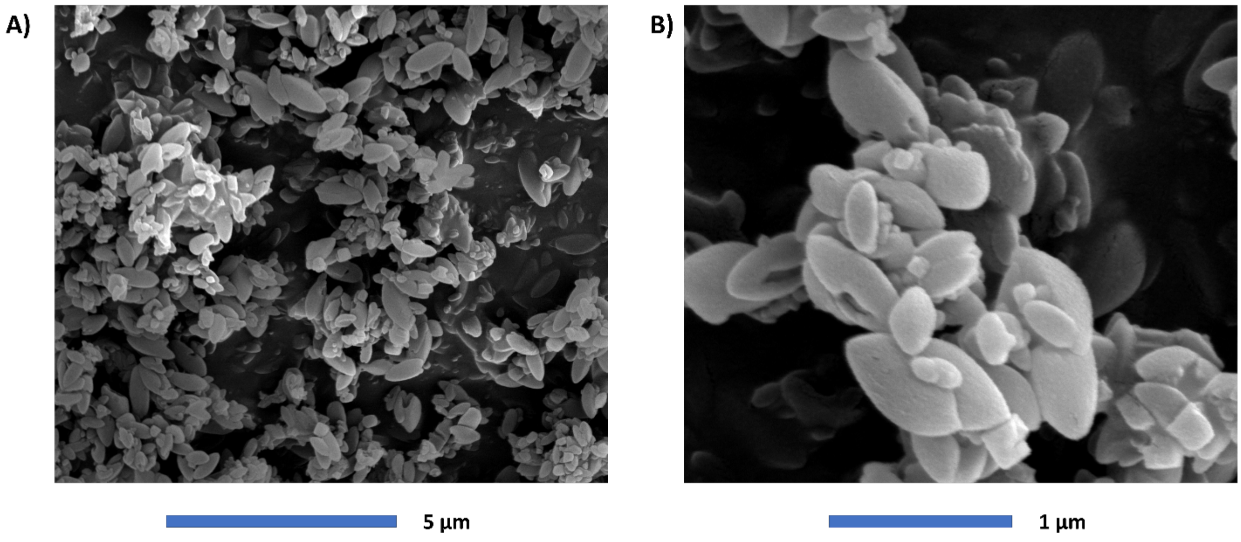

Figure S10: SEM images for I 65 °C.

Section S4: Crystallography data

Table S1. Data statistics. All datasets were collected at room temperature, using a wavelength of 0.0251 Å, scan width of 0.5° and exposure time of 1 s. A resolution cut-off was applied at 1.00 Å. Data set 'Merged' was obtained by merging of datasets 1 and 2. Point group symmetry: I 4/m.

Set	#frames	Time (min)	Completeness (%)	Redundancy	$<F^2/\sigma(F^2)>$	R_{int}	R_{pim}	R_{sigma}
**1**	180	3:10	99.7	3.5	7.65	0.155	0.102	0.101
**2**	200	3:30	89.4	4.2	10.98	0.150	0.087	0.078
**Merged**			99.7	7.2	10.89	0.206	0.086	0.071

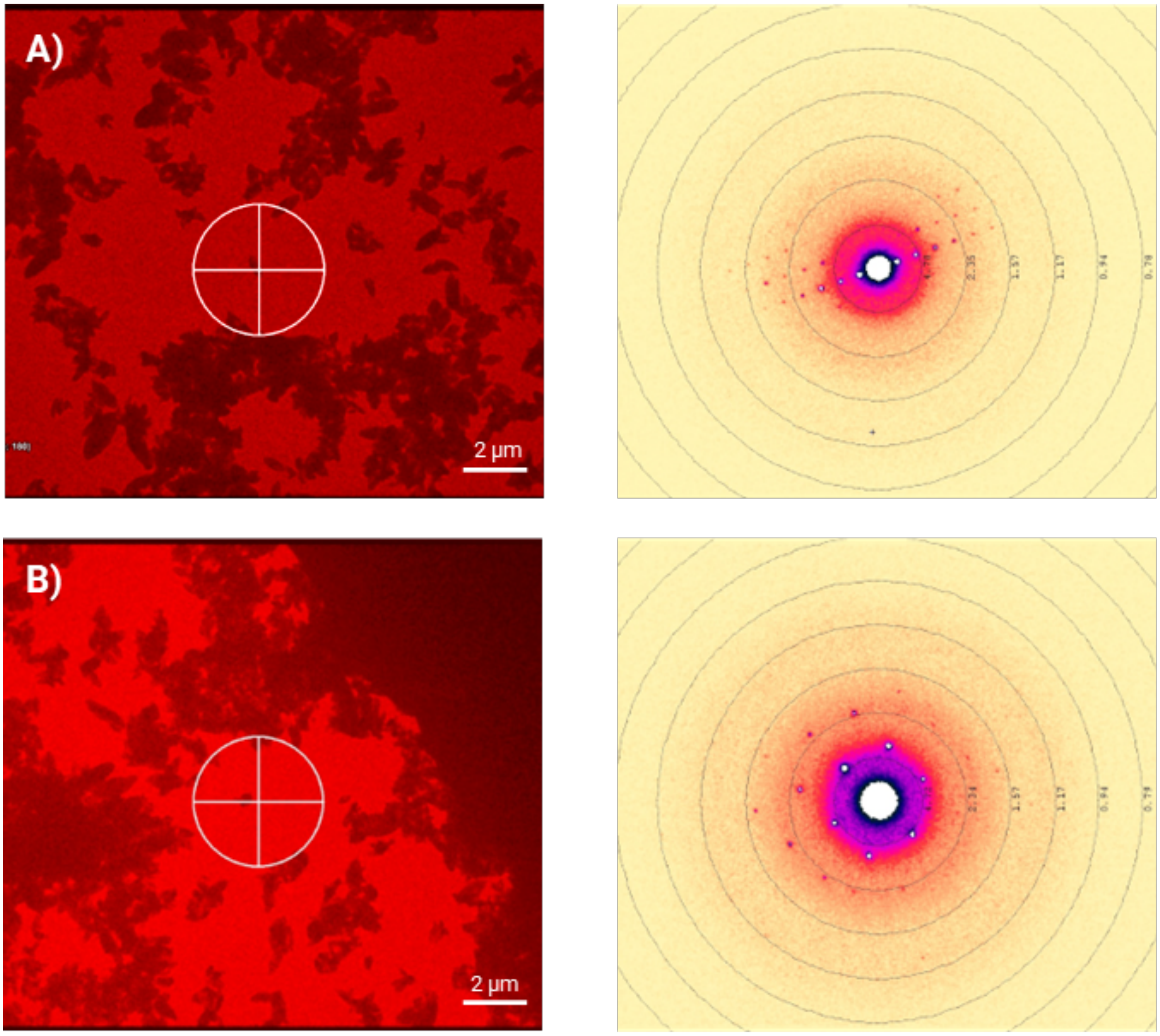

Figure S11: Grain and diffraction snapshots of the measured crystals: A) Crystal used for dataset 1, B) Crystal used for dataset 2 (**Table S1**).

Table S2. Data collection statistics vs resolution for the final dataset (Merged).

Resolution (Å)	Data	Completeness (%)	Redundancy	<F^2>	<F^2/σ(F^2)>	R_{int}	R_{pim}	R_{sigma}
Inf- 2.18	562	97.2	5.4	2245.95	45.28	0.076	0.040	0.016
2.18- 1.74	684	100	6.4	1144.36	29.68	0.129	0.058	0.042
1.74- 1.51	754	100	7.0	388.95	12.97	0.245	0.096	0.104
1.51- 1.37	797	100	7.4	157.63	6.05	0.384	0.151	0.207
1.37- 1.27	775	100	7.2	183.27	6.06	0.380	0.151	0.198
1.27- 1.20	789	100	7.4	253.81	7.98	0.338	0.134	0.163
1.20- 1.13	825	100	7.7	141.15	4.75	0.463	0.179	0.264
1.13- 1.08	812	100	7.6	107.98	4.07	0.528	0.207	0.363
1.08- 1.03	848	100	7.9	97.62	3.96	0.517	0.195	0.372
1.03- 1.00	819	100	7.6	46.21	1.87	0.694	0.270	0.576
**Inf- 1.00**	**7665**	**99.7**	**7.2**	**408.47**	**10.89**	**0.206**	**0.086**	**0.071**

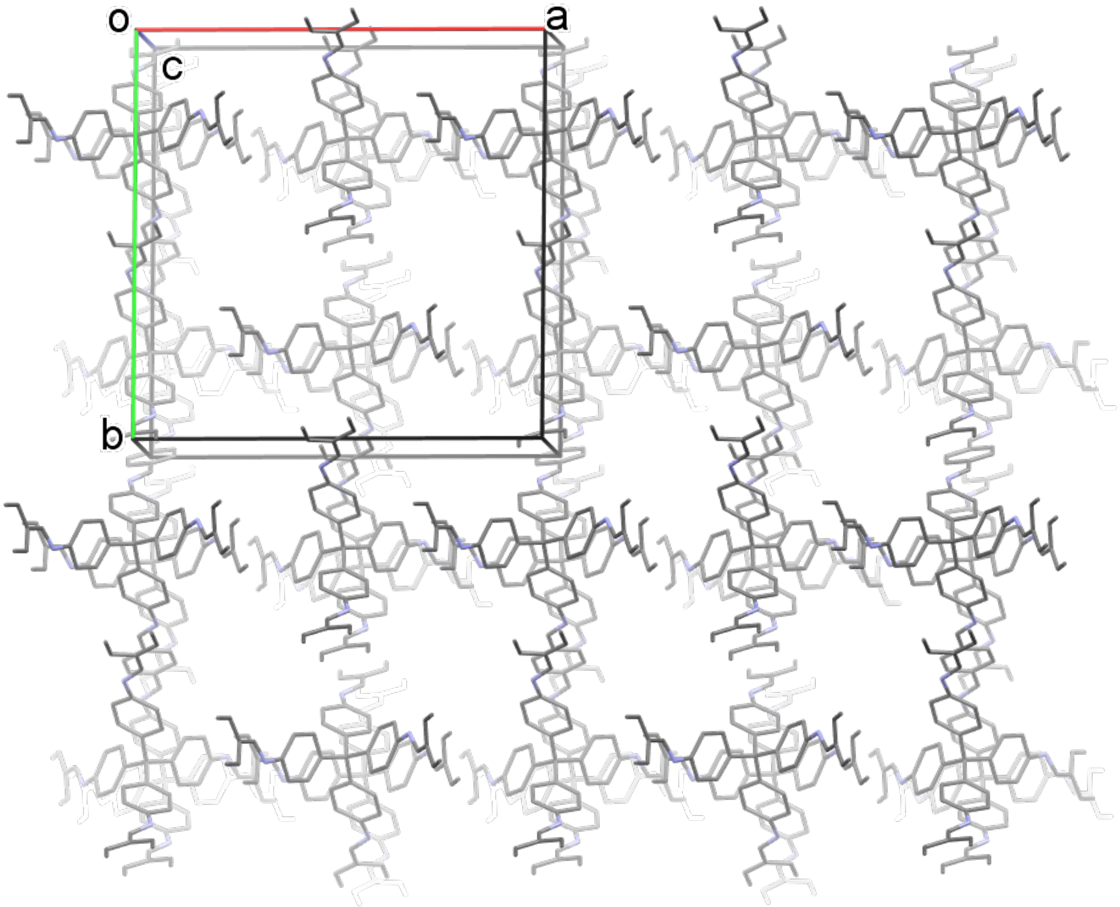

Figure S12: Crystal packing of COF-300 visualized using a capped sticked model with depth cueing. Hydrogens are omitted for clarity. The unit cell is indicated.

Table S3. Comparison of 3D ED data parameters obtained for different COFs.

Material	Temperature (K)	Resolution (Å)	Completeness (%)	R_{int}	Final R [I > 2σ(I)]	Parameters /Restraints	Ref
COF-320	89	1.5	97.5	0.481	R_1 = 0.3100, wR_2 = 0.6386	21/31	[5]
3D-TPB-COF-H	99	1.0	Not reported	0.331	R_1 = 0.3213, wR_2 = 0.6315	80/4	[6]
3D-TPB-COF-Me	99	0.90	Not reported	0.320	R_1 = 0.2897, wR_2 = 0.5952	80/4	[6]
3D-TPB-COF-F	99	1.0	Not reported	0.373	R_1 = 0.3133, wR_2 = 0.6049	80/5	[6]

COF-300-V	Room temperature[a]	1.0	35	0.141	R_1 = 0.2801, wR_2 = 0.5808	Not reported	[7]
COF-300-V	Cryo[a]	0.9	50	0.303	R_1 = 0.3175, wR_2 = 0.6295	Not reported	[7]
COF-300-H_2O	Cryo[a]	0.9	79	0.298	R_1 = 0.2911, wR_2 = 0.6187	Not reported	[7]
COF-300-IL	Cryo[a]	1.8	32	0.645	R_1 = 0.2513, wR_2 = 0.5311	Not reported	[7]
COF-300-PMMA	Cryo[a]	1.5	57	0.274	R_1 = 0.4033, wR_2 = 0.7525	Not reported	[7]
Py-1P	Room temperature[a]	0.9	41.8	0.099	R_1 = 0.135, wR_2 not reported	185/Not reported	[8]
COF-320-Micelle	100	0.9	99.7	0.2391	R_1 = 0.3053, wR_2 = 0.6589	Not reported	[9]
COF-904	Not reported	0.80	63.2	0.1843	R_1 = 0.2091, wR_2 = 0.4924	239/27	[10]
USTB-20-dia	100	0.9	Not reported	0.1577	R_1 = 0.1177, wR_2 = 0.2940	557/50	[11]
COF-300-V	100	0.85	99.0	0.1364	R_1 = 0.1684, wR_2 = 0.4430[b]	130/0	[12]
COF-300-H2O	100	0.80	97.2	0.1629	R_1 = 0.1612, wR_2 = 0.4324[b]	147/4	[12]
COF-300-CH3OH	100	0.85	97.7	0.1478	R_1 = 0.1601, wR_2 = 0.4518[b]	147/9	[12]
COF-300-C2H5OH	100	0.90	98.8	0.3682	R_1 = 0.1849, wR_2 = 0.5235[b]	139/9	[12]
COF-320-A	293	0.9	70.2	0.0912	R_1 = 0.178, wR_2 not reported	117/Not reported	[13]
COF-320-A2	293	1.0	91.6	0.224	R_1 = 0.204, wR_2 not reported	53/Not reported	[13]
3D-An-COF	77	1.1	Not reported	0.1632	R_1 = 0.2777, wR_2 = 0.6243	44/Not reported	[14]
COF-300	293	1.0	99.7	0.2204	R_1 = 0.1372, wR_2 = 0.3425	103/81	This work

a) No exact temperatures are given, only indication of cryo and room temperature.
b) R values obtained for kinematical refinement are listed for fair comparison.

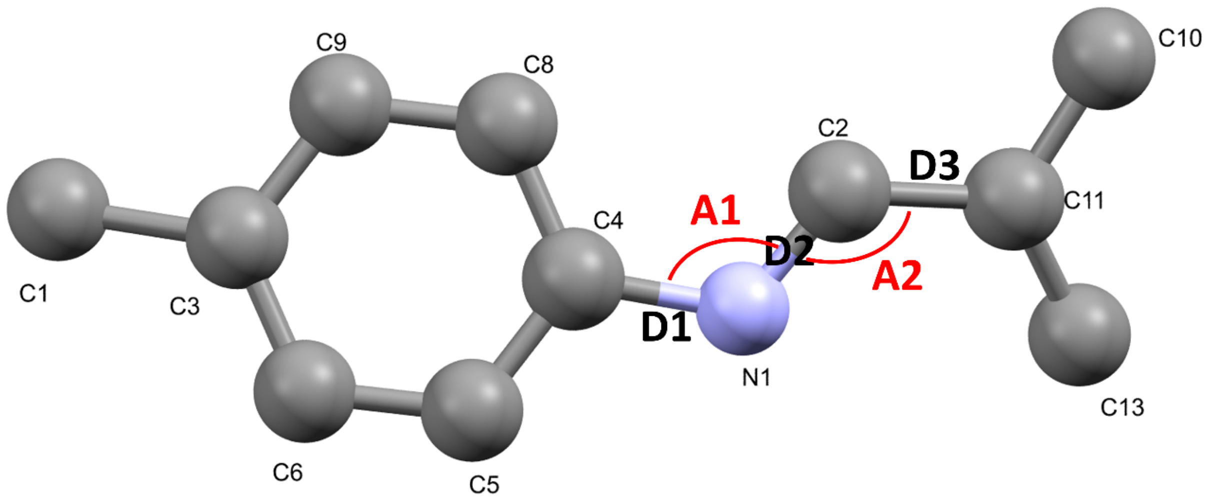

Figure S13: Ball and stick model of COF300_hydrated showing the labeling used in the original report.[15] Hydrogens atoms and solvent molecules are omitted for clarity.
Studied distances D1 (C4-N1), D2 (N1-C2) and D3 (C2-C11) are shown in black, studied angles A1 (C4-N1-C2) and A2 (N1-C2-C11) in red.

Table S4. Comparison of Bond lengths between COF300_hydrated and COF-300. Significant deviations are highlighted in yellow. Labelling is kept equal to the used labelling of the COF300_hydrated report[15] and presented in **Figure S11**.

SXRD (COF300_hydrated)			3D ED (COF-300)		
**Atom**	**Atom**	**Length/Å**	**Atom**	**Atom**	**Length/Å**
C1	C3	1.543(4)	C1	C3	1.55(2)
C1	C3[1]	1.543(4)	C1	C3[5]	1.53(2)
C1	C3[2]	1.543(4)	C1	C3[6]	1.53(2)
C1	C3[3]	1.543(4)	C1	C3[7]	1.53(2)
C3	C6	1.390(5)	C3	C6	1.34(2)
C3	C9	1.393(5)	C3	C9	1.41(2)
C4	C5	1.375(6)	C4	C5	1.37(2)
C4	C8	1.394(6)	C4	C8	1.40(3)
C4	N1	1.428(5)	C4	N1	1.41(3)
C5	C6	1.389(5)	C5	C6	1.41(3)
C8	C9	1.377(6)	C8	C9	1.40(3)
C10	C11	1.388(8)	C10	C11	1.40(3)
C10	C13[4]	1.395(6)	C10	C13[8]	1.41(4)
C11	C13	1.388(7)	C11	C13	1.39(3)
C11	C2	1.486(6)	C11	C2	1.48(4)

C13	C10[4]	1.395(6)	C13	C10[8]	1.41(4)
C2	N1	1.272(6)	C2	N1	1.25(2)

[1]1-X,1/2-Y,+Z; [2]1/4+Y,3/4-X,11/4-Z; [3]3/4-Y,-1/4+X,11/4-Z; [4]1-X,1-Y,1-Z ; [5]- 1-X,1/2-Y,+Z; [6]- 1/4+Y,3/4-X,11/4-Z; [7]- 3/4-Y,-1/4+X,11/4-Z; [8] 1-X,1-Y,1-Z

Table S5. Comparison of Bond angles between COF300_hydrated and COF-300. Significant deviations are highlighted in yellow. Labelling is kept equal to the used labelling of the COF300_hydrated report[15] and presented in **Figure S11**.

SXRD (COF300_hydrated)				3D ED (COF-300)			
**Atom**	**Atom**	**Atom**	**Angle/°**	**Atom**	**Atom**	**Atom**	**Angle/°**
C3	C1	C3[1]	101.5(3)	C3	C1	C3[5]	102.6(19)
C3[1]	C1	C3[2]	113.58(13)	C3	C1	C3[7]	113.0(10)
C3	C1	C3[2]	113.58(14)	C3[5]	C1	C3[6]	113.0(10)
C3[1]	C1	C3[3]	113.57(14)	C3[5]	C1	C3[7]	113.0(10)
C3	C1	C3[3]	113.58(13)	C3[6]	C1	C3	113.0(10)
C3[2]	C1	C3[3]	101.5(3)	C3[5]	C1	C3[7]	102.6(19)
C6	C3	C1	122.8(3)	C6	C3	C1	123.4(8)
C6	C3	C9	118.1(3)	C6	C3	C9	118.7(12)
C9	C3	C1	117.9(3)	C9	C3	C1	116.3(11)
C5	C4	C8	118.9(4)	C5	C4	C8	117.4(13)
C5	C4	N1	118.0(4)	C5	C4	N1	117.2(13)
C8	C4	N1	122.9(4)	C8	C4	N1	124.9(10)
C4	C5	C6	121.0(4)	C4	C5	C6	120.3(14)
C5	C6	C3	120.4(4)	C3	C6	C5	122.4(10)
C9	C8	C4	120.1(4)	C4	C8	C9	122.3(10)
C8	C9	C3	121.4(4)	C3	C9	C8	118.8(13)
C11	C10	C13[4]	120.4(5)	C11	C10	C13[8]	119.1(15)
C10	C11	C13	120.6(4)	C13	C11	C10	119.4(17)
C10	C11	C2	117.2(5)	C10	C11	C2	119.5(14)
C13	C11	C2	121.9(5)	C13	C11	C2	120.7(13)
C11	C13	C10[4]	119.1(5)	C11	C13	C10[8]	121.5(14)
N1	C2	C11	122.6(5)	N1	C2	C11	123.6(14)
C2	N1	C4	118.1(4)	C2	N1	C4	119.1(14)

[1]1-X,1/2-Y,+Z; [2]1/4+Y,3/4-X,11/4-Z; [3]3/4-Y,-1/4+X,11/4-Z; [4]1-X,1-Y,1-Z [5]-X,1/2-Y,+Z; [6]1/4-Y,1/4+X,1/4-Z; [7]-1/4+Y,1/4-X,1/4-Z; [8]-1/2-X,1/2-Y,-3/2-Z

Table S6. Zoom in on the formed imine connection as encountered in COF-300 (phenyl-N=CH-phenyl). SXRD structure (COF300_hydrated) and 3D ED (COF-300) structure are compared to mean values as found in CSD database.[16] Studied distances and angles are presented in **Figure S12**.

	**CSD Data[a]**	**SXRD (COF300_hydrated)**	**3D ED (COF-300)**
D1 (Å)	1.427 (16)	1.428 (5)	1.41 (3)
D2 (Å)	1.288 (30)	1.272 (6)	1.25 (2)

D3 (Å)	1.478 (18)	1.486 (6)	1.48 (4)
θ1 (°)	120.830 (2867)	118.1 (4)	119.1 (15)
θ2 (°)	122.735 (3232)	122.6 (5)	123.6 (15)

[a] Mean bond lengths and angles based on Conquest search[17] with a (phenyl-N=CH-phenyl) query. Mean values and standard deviations based on 438 hits and 580 different imine bonds were used.

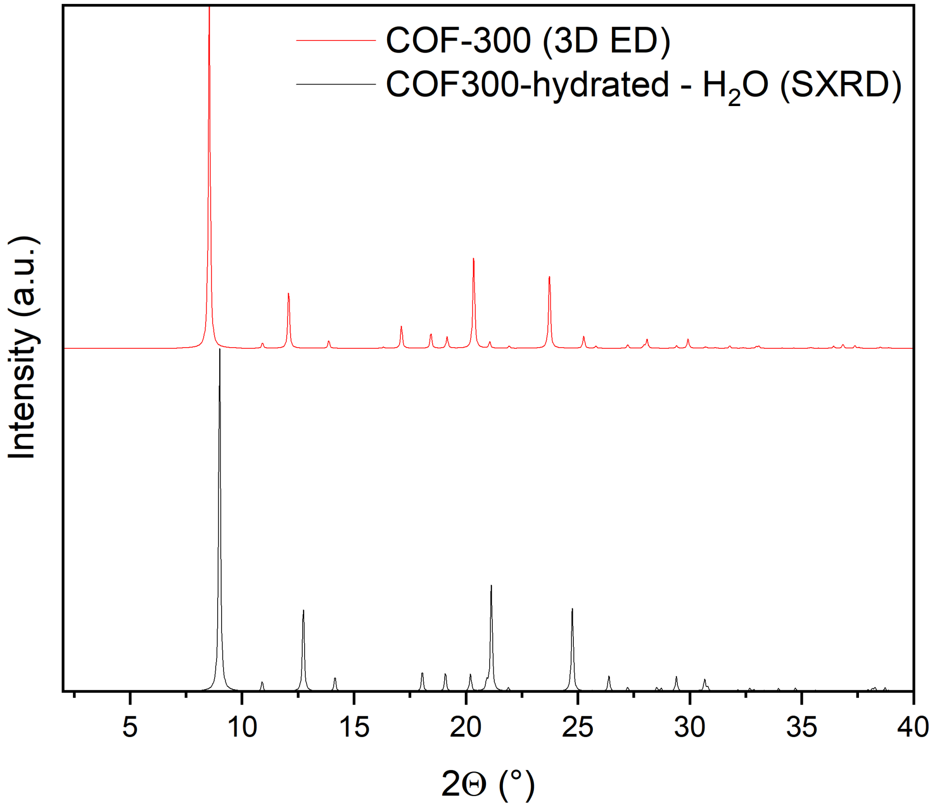

Figure S14: Comparison of simulated PXRD patterns obtained from synchotron SXRD[15] (COF-300 hydrated, black) and 3D ED (COF-300, red). For proper comparison, H_2O molecules were removed from the COF-300 hydrated structure. The shift to lower angles observed for the 3D ED structure can be explained by the expanded unit cell, as a consequence of RT data collection.

Section S5: references

1) Rigaku Oxford Diffraction, CrysAlisPro Software system, Rigaku Corporation, Yarnton, UK, 2022.
2) O. V. Dolomanov , L. J. Bourhis , R. J. Gildea , J. A. K. Howard, H. Puschmann , *J. Appl. Crystallogr.*, **2009**, 42 , 339-34.
3) G. M. Sheldrick *Acta Crystallogr., Sect. A: Found. Crystallogr.*, 2015, **71**, 3-8.
4) G. M. Sheldrick *Acta Crystallogr., Sect. C: Struct. Chem.*, 2015, **71**, 3-8.

5) Y.-B. Zhang, J. Su, H. Furukawa, Y. Yun, F. Gándara, A. Duong, X. Zou and O. M. Yaghi, *Journal of the American Chemical Society*, 2013, **135**, 16336-16339.
6) C. Gao, J. Li, S. Yin, G. Lin, T. Ma, Y. Meng, J. Sun and C. Wang, *Angewandte Chemie International Edition*, 2019, **58**, 9770-9775.
7) T. Sun, L. Wei, Y. Chen, Y. Ma and Y.-B. Zhang, *Journal of the American Chemical Society*, 2019, **141**, 10962-10966.
8) C. Kang, K. Yang, Z. Zhang, A. K. Usadi, D. C. Calabro, L. S. Baugh, Y. Wang, J. Jiang, X. Zou, Z. Huang and D. Zhao, *Nature Communications*, 2022, **13**, 1370.
9) Z. Zhou, L. Zhang, Y. Yang, I. J. Vitorica-Yrezabal, H. Wang, F. Tan, L. Gong, Y. Li, P. Chen, X. Dong, Z. Liang, J. Yang, C. Wang, Y. Hong, Y. Qiu, A. Gölzhäuser, X. Chen, H. Qi, S. Yang, W. Liu, J. Sun and Z. Zheng, *Nature Chemistry*, 2023, **15**, 841-847.
10) Y. Xiao, Y. Ling, K. Wang, S. Ren, Y. Ma and L. Li, *Journal of the American Chemical Society,* 2023, **145**, 13537-13541.
11) B. Yu, W. Li, X. Wang, J.-H. Li, R.-B. Lin, H. Wang, X. Ding, Y. Jin, X. Yang, H. Wu, W. Zhou, J.Zhang and J. Jiang, *Journal of the American Chemical Society*, 2023, **145**, 25332-25340.
12) W. Sun, P. Chen, M. Zhang, J. Ma and J. Sun, *Angewandte Chemie International Edition*, 2023, **62**, e202305985.
13) C. Kang, Z. Zhang, S. Kusaka, K. Negita, A. K. Usadi, D. C. Calabro, L. S. Baugh, Y. Wang, X. Zou, Z. Huang, R. Matsuda and D. Zhao, *Nature Materials*, 2023, **22**, 636-643.
14) Y. Cheng, J. Xin, L. Xiao, X. Wang, X. Zhou, D. Li, B. Gui, J. Sun and C. Wang, *Journal of the American Chemical Society*, 2023, **145**, 18737-18741.
15) T. Q. Ma, E. A. Kapustin, S. X. Yin, L. Liang, Z. Y. Zhou, J. Niu, L. H. Li, Y. Y. Wang, J. Su, J. Li, X. G. Wang, W. D. Wang, W. Wang, J. L. Sun and O. M. Yaghi, *Science*, 2018, **361**, 48-52.
16) C. R. Groom, I. J. Bruno, M. P. Lightfoot and S. C. Ward, *Acta Crystallographica Section B Structural Science, Crystal Engineering and Materials*, 2016, **72**, 171-179.
17) I. J. Bruno, J. C. Cole, P. R. Edgington, M. Kessler, C. F. Macrae, P. McCabe, J. Pearson and R. Taylor *Acta Cryst. B*, 2002, **58**, 389-397.
